# Synthesis and Coordination Properties of a Water-Soluble Material by Cross-Linking Low Molecular Weight Polyethyleneimine with Armed Cyclotriveratrilene

**DOI:** 10.3390/polym13234133

**Published:** 2021-11-26

**Authors:** Yoke Mooi Ng, Paolo Coghi, Jerome P. L. Ng, Fayaz Ali, Vincent Kam Wai Wong, Carmine Coluccini

**Affiliations:** 1 Institute of New Drug Development, College of Medicine, China Medical University, No. 91 Hsueh-Shih Road, Taichung 40402, Taiwan; yumei.wu91@gmail.com; 2 School of Pharmacy, Macau University of Science and Technology, Macau 999078, China; coghips@must.edu.mo; 3 Neher’s Biophysics Laboratory for Innovative Drug Discovery, State Key Laboratory of Quality Research in Chinese Medicine, Macau University of Science and Technology, Macau 999078, China; plng@must.edu.mo (J.P.L.N.); bowaiwong@gmail.com (V.K.W.W.); 4 Department Chemistry, Abbottabad University of Science and Technology, Abbottabad 22010, Pakistan; fayazalisabir@gmail.com

**Keywords:** polyethyleneimine, cyclotriveratrilene, polymer cross-linking, hydrophilic material, hydrophobic interaction

## Abstract

In this study, a full organic and water-soluble material was synthesized by coupling low molecular weight polyethylenimine (PEI-800) with cyclotriveratrilene (CTV). The water-soluble cross-linked polymer contains hydrophobic holes with a high coordination capability towards different organic drug molecules. The coordinating capability towards hydrophilic drugs (doxorubicin, gatifloxacin and sinomenine) and hydrophobic drugs (camptothecin and celastrol) was analyzed in an aqueous medium by using NMR, UV-Vis and emission spectroscopies. The coordination of drug molecules with the armed CTV unit through hydrophobic interactions was observed. In particular, celastrol exhibited more ionic interactions with the PEI moiety of the hosting system. In the case of doxorubicin, the host–guest detachment was induced by the addition of ammonium chloride, suggesting that the intracellular environment can facilitate the release of the drug molecules.

## 1. Introduction

In the past 60 years, scientists have been inspired by biology concepts to prepare artificial systems by assembling different molecules with physical forces [1,2,3]. The process of replicating enzyme–substrate interactions, known as molecular recognition, entails the production of large molecules or multimolecular entities, as well as the examination of the hosting capability towards relatively tiny charged or neutral small molecules [4,5].

The requirement of limiting or rendering more discriminant drug toxicity interests researchers in preparing and investigating different drug-coordinating platforms [6,7,8]. Polymers have been found to have intensive applications for coordination with therapeutic agents due to their large molecular structures and functionalization [9].

Polyethyleneimine (PEI) consists of a mixture of primary, secondary and tertiary amines that can be protonated over a wide pH range and modified by exploiting the reactivity of the amino group [10,11]. Of note, the original or modified form of PEI mainly concerned the gene coordination due to the positive charges that the polymer exhibits in a water solution upon amino groups’ protonation [10,12,13]. Meanwhile, PEI is often conjugated to small aromatic molecules, which improves the cell transfection efficiency or facilitates the fluorescent polymeric nanoparticles preparation [14,15]. For instance, doxorubicin (DOX) can be linked to PEI through covalent connection or coordinated through supramolecular interactions for drug-delivery applications [16,17]. Our group previously also reported the coordination of doxorubicin with PEI-25k cross-linked cyclotriveratrylene (CTV) [18].

**Figure 1:**
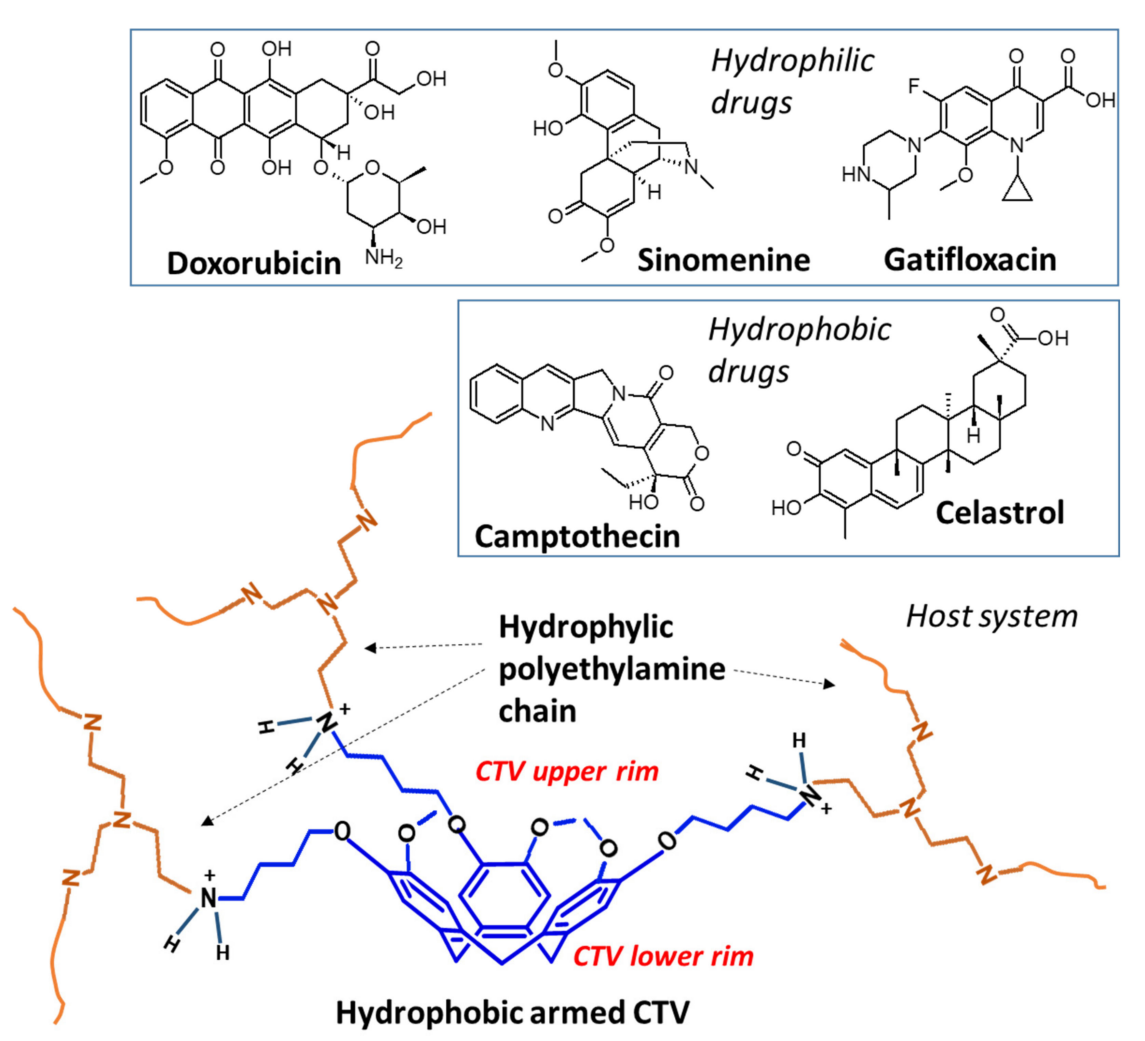
Illustration of the synthesized coordinating system and molecular structures of drug molecules analyzed in this study.

CTV is a cyclic trimer with a rigid conformation [19]. The most stable configuration of CTV exhibits a distance between the centers of the rings of ~4.77 Å, and the cavity dimension may vary depending on the length of appended side arm to the upper rim (Figure 1) [20]. The concave-shaped cavity is responsible for the vast variety of supramolecular interactions with ionic and neutral small molecules [21,22,23,24,25,26,27,28,29,30,31,32,33]. Moreover, armed CTV can even coordinate large structures such as fullerenes, showing high flexibility towards different sizes of molecules [32,33,34,35,36,37,38,39,40,41,42].

Theoretically, the combination of CTV and PEI may provide a viable delivery platform for medicines with varied characteristics. The choice of the small molecular weighted polymer can induce drastic changes in the physical properties and coordinating capability, and therefore we intend to combine cyclotriveratrilene with **PEI-800** for developing the platform.

The new cross-linked polymer (**Cross-PEI-800**) exhibited complete solubility in water, which facilitated the examination of the polymer itself in terms of its interactions with other compounds and cell viability. In addition, we also analyzed the coordination of **Cross-PEI-800** with five different selected drug molecules to improve their water solubility: hydrophilic drugs—doxorubicin [43,44,45], gatifloxacin [46] and sinomenine [47]; hydrophobic drugs—camptothecin [48] and celastrol [49,50,51].

Formulations of the abovementioned drugs for selective targeting envisage two main strategies: chemical modifications and supramolecular encapsulation [52,53,54,55,56,57,58,59,60,61,62,63]. Supramolecular coordination is often preferable over other interactions due to its better external stimuli response, which modulates the coordination and releasing processes [63,64].

Although the selected compounds possess different physical properties, they exhibit similar structural features, including π-conjugated systems and heteroatoms. We herein developed a drug delivery platform, as shown in Figure 1, by exploiting these features for supramolecular coordination. The hydrophobic rigid CTV cup is suitable for interacting with the π-conjugated system of those molecules, and meanwhile, polyethyleneimine chains are more inclined to hydrogen bond coordination with the heteroatoms.

## 2. Materials and Methods

### 2.1. General

Human lung adenocarcinoma (A549), immortalized normal human hepatocytes (LO2), and human embryonic kidney cells (HEK293) were bought from ATCC (ATCC, Manassas, VA, USA). Cells were incubated in RPMI-1640 (A549 cells) and DMEM (LO2 and HEK293 cells) media, which contain 10% fetal bovine serum and antibiotics (penicillin, 50 U/Ml, and streptomycin, 50 mg/mL; Invitrogen, Paisley, Scotland, UK). All cells were cultured at 37 °C in a 5% humidified CO_2_ incubator. Stock solutions of all tested compounds were prepared by dissolving in DMSO and stored at −20 °C before use.

### 2.2. Cytotoxicity Assay

Cell viability was determined by the 3-[4,5-dimethylthiazole-2-yl]-2,5-diphenyltetrazolium bromide (MTT) assay as reported previously [65]. Cells with a density of 5 × 10^3^ per well were seeded in 96-well plates before treatment of drugs. After overnight culture, cells were treated with different concentrations of each compound (0.191–100 μM) for 72 h. Cells without drug treatment were used as controls. After that, 10 μL of MTT solution (5 mg/mL) was added to the wells and incubated at 37 °C for another 4 h. Finally, 100 μL of solubilization buffer (12 mM of HCl in 10% SDS) was added to each well and incubated overnight. The absorbance of each well was then determined at 570 nm the next day. The percentage of cell viability was calculated, and the cytotoxicity was presented as % Viability = A_treated_/A_control_ × 100.

### 2.3. Materials

Vanillyl alcohol, 1,4-dibromobutane, scandium triflate Sc(OTf)_3_ and branched polyethyleneimine (**PEI-800**) were purchased from Sigma-Aldrich (average Mw 800 by LS, average Mn 600 by GPC, branched) and used without further purification. Unless specified otherwise, acetone and acetonitrile (CH_3_CN) were used as acquired from commercial sources. The dialysis bag for the purification of polymer was Spectra/Por^®^6 Dialysis Membrane; MWCO: 1 kD. ^1^H and ^13^C NMR spectra were recorded on a Bruker Avance III 400 MHz BBFO Probe and AVANCE NEO 400 NMR in CDCl_3_, D_2_O and DMSO-*d*_6_, using the solvent residual proton signal as a reference. Flash column chromatography was performed using Silicycle Silica gel 60 (7–230 Mesh, pH 7). UV-Vis absorption spectra of all samples in H_2_O were obtained by Agilent Cary 60UV–Visible, Thermo Scientific Multiskan G0 microplate and cuvette spectrophotometer over a range of 200–1000 nm. Fluorescence spectra were acquired using Perkin Elmer, LS50B Luminescence Spectrometer equipped with an SC-05 standard cuvette holder.

### 2.4. Synthesis

**[4-(2-bromobutoxy)-3-methoxyphenyl]methanol (2a):** To a solution of 3-methoxy-4-hydroxybenzylalcohol **1a** (2 g, 13 mmol, 1 equiv.) in acetone (24 mL), K_2_CO_3_ (8.97 g, 65 mmol, 5 equiv.) was added and stirred for 30 min at room temperature under argon. After addition of 1,4-dibromobutane (15.5 mL, 130 mmol, 10 equiv) in one portion, the resulting mixture was heated at 55 °C overnight. The reaction mixture was quenched with water (160 mL), extracted with ethyl acetate (3 × 80 mL) and concentrated in vacuo to obtain a yellow oil as the crude product. The crude product was purified on silica gel using ethyl acetate/hexane 4:6 as eluent to afford **2a** (2.42 g, 8.40 mmol, 64%) as a white solid. ^1^H-NMR (CDCl_3_, 400 MHz, 25 °C): δ 6.92 (m, 1H), 6.86 (m, 1H), 4.62 (m, 2 H), 4.05 (t, J = 6.3 Hz, 2H), 3.88 (s, 3H), 3.50 (t, J = 6.35 Hz, 2H), 2.07 (m, 2H), 2.00 (m, 2H); ^13^C-NMR (CDCl_3_, 100 MHz, 25 °C): δ 149.5, 147.9, 134.0, 131.2, 119.9, 112.5, 110.5, 68.1, 65.2, 56.3, 33.5, 29.4, 27.8 ppm.

**2,7,12-Tris-(2-bromobutoxy)-3,8,13-trimethoxy-10,15-dihydro-2H-tribenzo[a,d,g]cyclononene (Armed CTV):** Sc(OTf)_3_ (0.114 g, 0.231 mmol, 0.030 equiv) was added to a solution of **2a** (2.22 g, 7.70 mmol, 1.0 equiv.) in dry CH_3_CN (50 mL) under nitrogen atmosphere and heated under reflux for 2 days. The solvent was removed in vacuo. The resulting mixture was redissolved in ethyl acetate (100 mL) and washed with water (2 × 200 mL). The combined organic layers were concentrated in vacuo to obtain a yellow oil as the crude product. The crude product was purified on silica gel using ethyl acetate/hexane 1:4 as eluent to afford the pure product as a white solid (983 mg, 1.21 mmol, 47%). ^1^H-NMR (CDCl_3_, 400 MHz, 25 °C): δ 6.85 (m, 6H), 4.76 (d, J = 14 Hz, 3H), 4.03 (m, 2H), 3.84 (s, 9H), 3.55 (d, J = 14 Hz, 3H), 3.49 (td, J1 = 6.24 Hz, J2 = 2.36 Hz, 6H), 2.07 (q, J = 5.84, 6H), 1.96 (q, J = 5.84, 6H); ^13^C-NMR (CDCl_3_, 100 MHz, 25 °C): δ 148.4, 147.1, 132.4, 131.9, 115.5, 113.9, 68.4, 56.3, 36.5, 33.5, 29.4, 27.9 ppm; MALDI-TOF mass [M]^+^ calc. 810.0766, exp. 810.0761, [M + Na]^+^ calc. 833.0658, exp. 833.0658.

**Synthesis of cross-linked polymer (Cross-PEI-800)**. Branched **PEI-800** (200 mg) was dissolved in 8 mL of dry DMSO and injected by a syringe in a dry round bottom flask containing **Armed CTV** (200 mg). The reaction solution was stirred at 75 °C for 5 days. After cooling to room temperature, the solution was transferred to dialysis bag 1000 Da Mw cut off, which was immersed in methanol for 24 h under magnetic stirring. During this time the methanol solution was changed three times. The solution in the bag was evaporated, and 228 mg of yellow gel was collected.

### 2.5. Spectroscopic Studies

**UV spectra of doxorubicin, gatifloxacin, sinomenine, camptothecin, pure and mixed with Cross-PEI-800.**

An amount of 1 mg of drug was dissolved in 10 mL of water, and the reported concentrations were obtained by sample dilution. The mixed solutions were prepared by the addition of **Cross-PEI-800** to the solution of pure drug.

#### 2.5.1. UV Spectra of Celastrol

Celastrol (1 mg, 2.2 × 10^−3^ mmol) was dissolved in water (1 mL) under argon. This solution (1 mL) was transferred into a UV cuvette under argon.

#### 2.5.2. UV Spectra Celastrol with Addiction of Solution of **Cross-PEI-800** (1%)

Celastrol (1 mg, 2.2 × 10^−3^ mmol) was dissolved in water (1 mL) under argon. This solution was added with 100 μL of solution to 10 mg/mL of **Cross-PEI-800** (1% in weight of solution). An amount of 1 mL of this solution was transferred into a UV cuvette under argon.

## 3. Results

### 3.1. Synthesis and Characterization

**Scheme 1:**
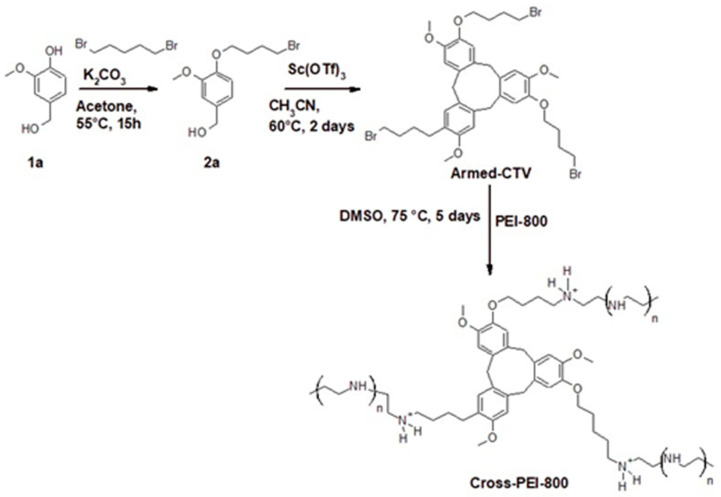
Synthesis of **Cross-PEI-800** by cross-linking **PEI-800** with Armed-CTV.

The cross-linked polymer **Cross-PEI-800** was prepared as shown in Scheme 1. Precursor **2a** was obtained by reacting vanillyl alcohol with 1,4-dibromobutane, as reported in the literature [18,66,67,68]. The oligomerization of compound **2a** with scandium triflate in CH_3_CN afforded **Armed-CTV** [18]. The **PEI-800** was cross-linked with **Armed-CTV** in DMSO overnight to obtain the highly water-soluble product **Cross-PEI-800**.

**Figure 2:**
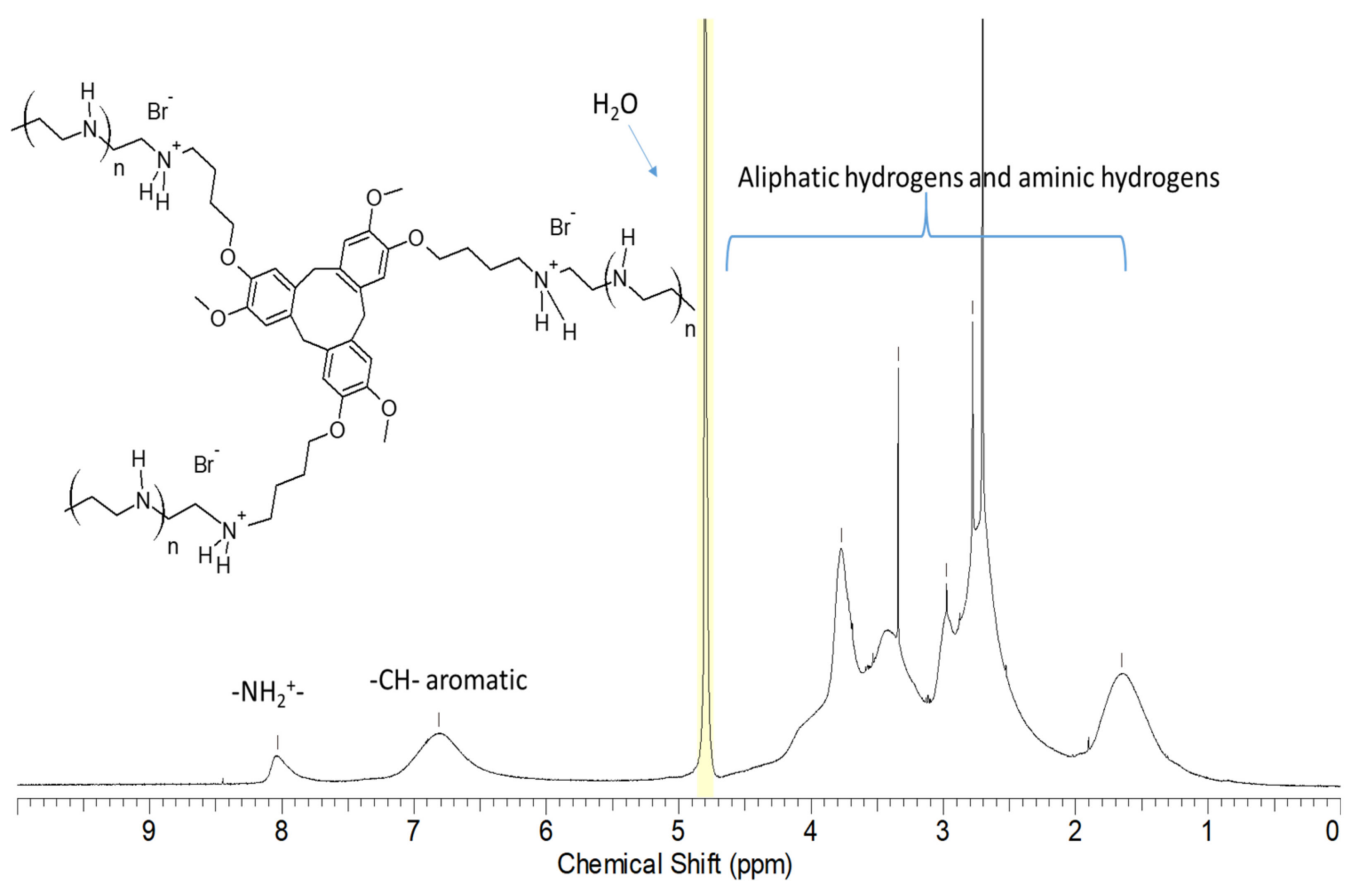
^1^H-NMR spectrum and peak assignments of **Cross-PEI-800** in D_2_O.

The ^1^H-NMR spectrum of **Cross-PEI-800** in D_2_O exhibited three broad peaks around 8 ppm, 7 ppm and 1–4 ppm, which were assigned (based on the NMR signals of the precursors) to the –NH_2_^+^-, aromatic –CH- and non-aromatic protons (aliphatic, -NH-, -OCH_n_-), respectively (Figure 2).

**Figure 3:**
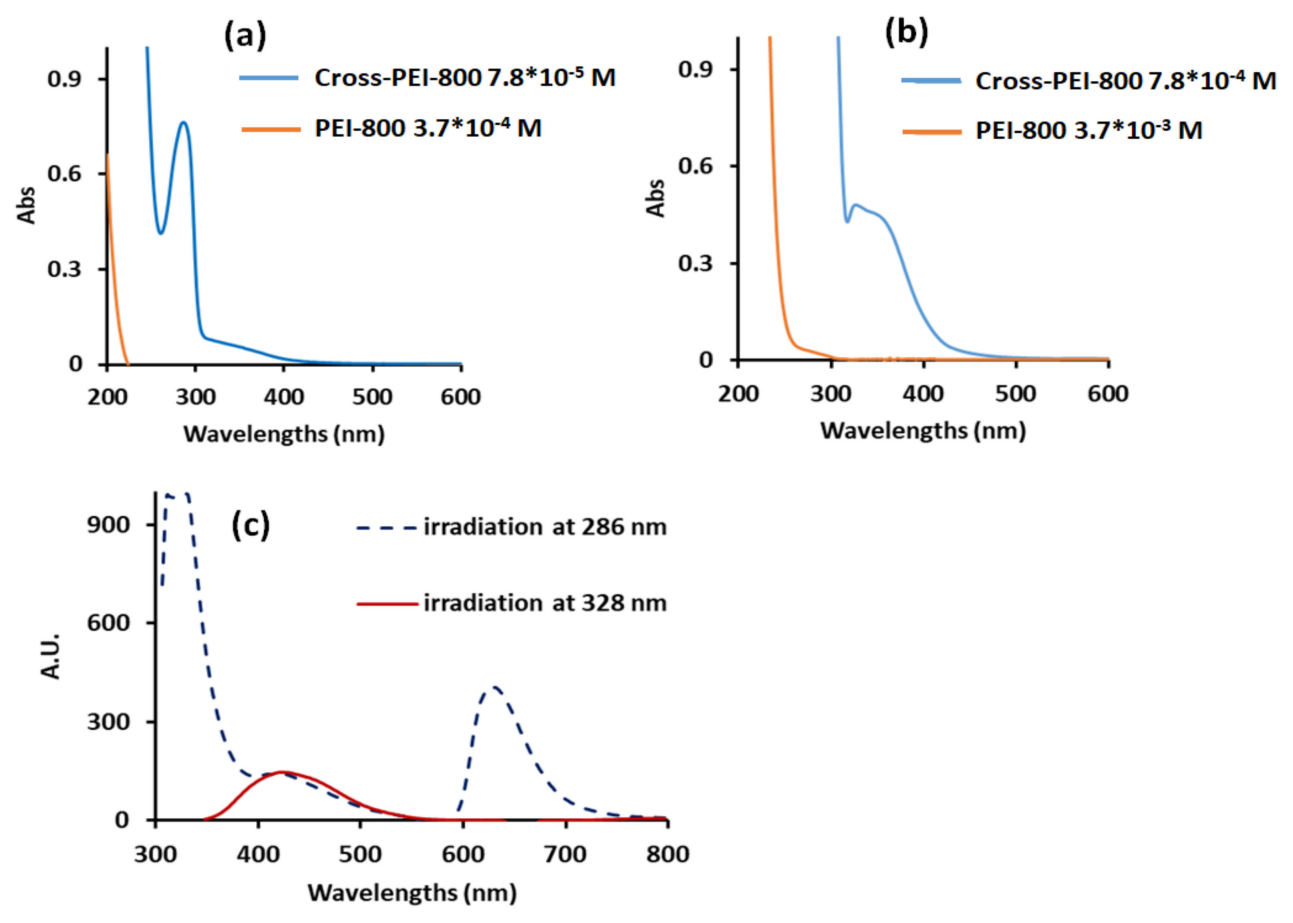
UV-Vis spectra in water of **Cross-PEI-800** at concentrations 7.8 × 10^−5^ M (**a**), 7.8 × 10^−4^ M (**b**) and emission spectrum at concentration 1.5 × 10^−4^ M (**c**). The concentration refers to the moles of monomers, whose molecular weight calculations are shown in the Supplementary Materials in the appendix of Figure S5.

**Cross-PEI-800** displayed UV-Vis spectra with absorption maxima at 286 and 328 nm, while **PEI-800** exhibited no absorption maxima (Figure 3a,b). Notably, **Cross-PEI-800** exhibited a better absorption band at 328 nm in a more concentrated solution. 

The irradiation of **Cross-PEI-800** solution at 286 nm produced a sharp emission at 330 nm and a broad emission at 450 and 630 nm, while the irradiation at 328 nm produced a broad emission at 450 nm (Figure 3c). Of note, the precursor **Armed-CTV** did not show any signals because of its insolubility in water.

The cytotoxicity of **Cross-PEI-800** was evaluated against the human normal cells (LO2 and HEK293) and human lung cancer cells (A549) using an MTT assay. **Cross-PEI-800** showed higher IC_50_ values against A549 and HEK293 cells than against LO2 cells [65].

The optical properties and the cell viability values of **Cross-PEI-800** are summarized in Table 1.

### 3.2. Drug Coordination Analysis

#### 3.2.1. Doxorubicin (Hydrophilic Drug)

**Figure 4:**
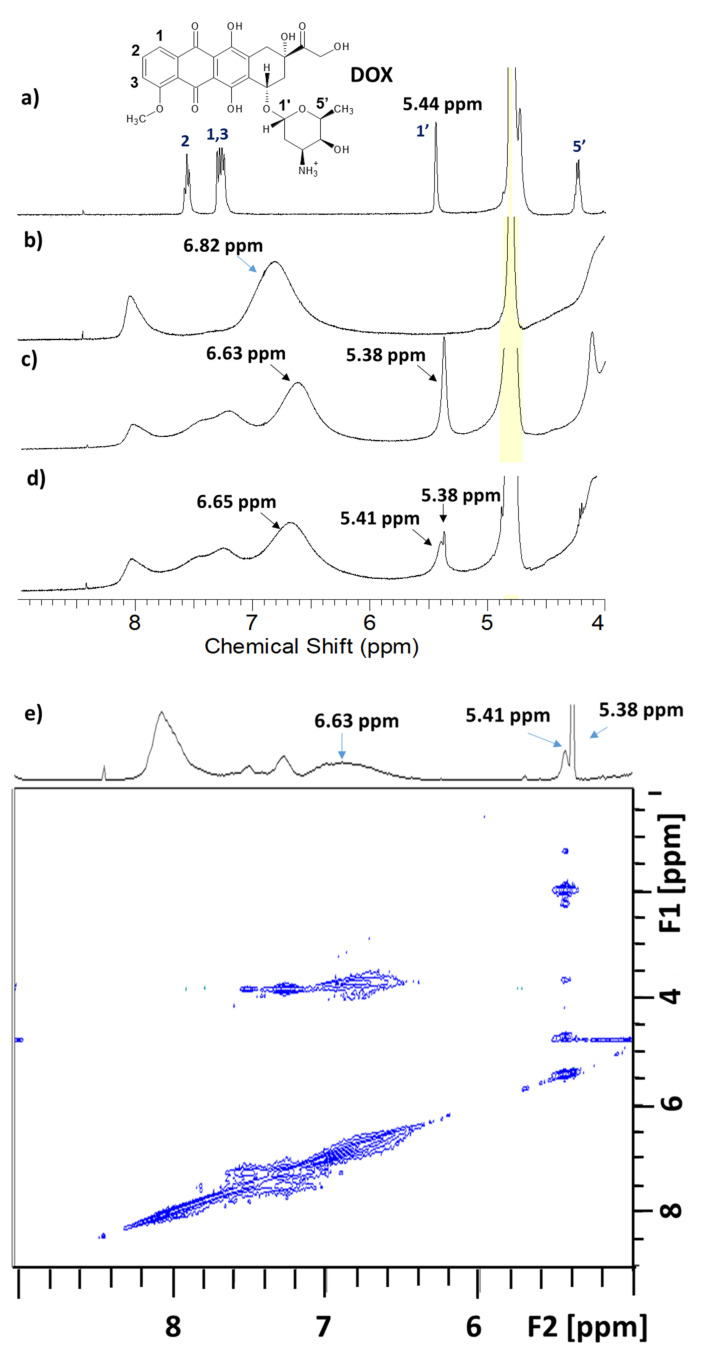
^1^H-NMR spectra, range 4–9 ppm of (**a**) Doxorubicin, (**b**) **Cross-PEI-800**, (**c**) **Cross-PEI-80**0 + DOX, (**d**) **Cross-PEI-800** + DOX after 7 days, and (**e**) NOESY spectrum of **Cross-PEI-800** + DOX after 7 days. The ppm full-range spectra shown in the Supplementary Materials (Figures S6–S9).

The ^1^H NMR spectra of DOX and the coordinating system (**Cross-PEI-800**) were obtained in D_2_O, and their corresponding NMR signals were assigned as reported in the literature (Figure 4a,b) [69]. After mixing **Cross-PEI-800** with DOX in D_2_O, the aromatic signals of the drug were broadened, and the aromatic signal of the polymer was shifted upfield by ~0.2 ppm (Figure 4c), showing a weak π−π stacking interaction between the cross-linked polymer and DOX phenyl rings. The broadening and shifting of the 1′ proton signal of DOX from 5.44 to 5.38 ppm were also observed (Figure 4c). After seven days, the broadened proton signal at 5.38 ppm was further split into two peaks at 5.38 and 5.41 ppm (Figure 4d). According to the NOESY spectrum, the peak at 5.41 ppm interacted with the aliphatic protons of DOX, while the signal at 5.38 ppm did not show intramolecular interactions except for the interaction with residual H_2_O (Figure 4e). Thus, the signal at 5.41 ppm corresponds to the 1′ proton of DOX, and the signal at 5.38 ppm corresponds to the –OH linked to the aliphatic carbons of the drug molecule (Figure 4d). In the solution of pure DOX, the –OH signal was not visible because of the proton exchange with D_2_O. Once the polymer was added, the hydroxyl proton was shielded from the interactions with solvent molecules and finally became detectable in ^1^H NMR spectrum. The ^1^H-NMR analysis (full 1HNMR spectra, Figures S6–S8 in the Supplementary Materials) unequivocally showed the hydrophobic interactions between **Cross-PEI-800** and DOX. In addition, the NOESY spectrum also revealed that the aromatic signals of the cross-linked polymer and drug molecules interacted with the signals at 3.8–4.1 ppm that correspond to –OMe and -OCH_2_- signals (full NOESY spectrum, Figure S9, Supplementary Materials). This demonstrates that the DOX located inside the hydrophobic environment of armed CTV is oriented with the –OMe group pointing towards the aromatic rings of the CTV unit.

**Figure 5:**
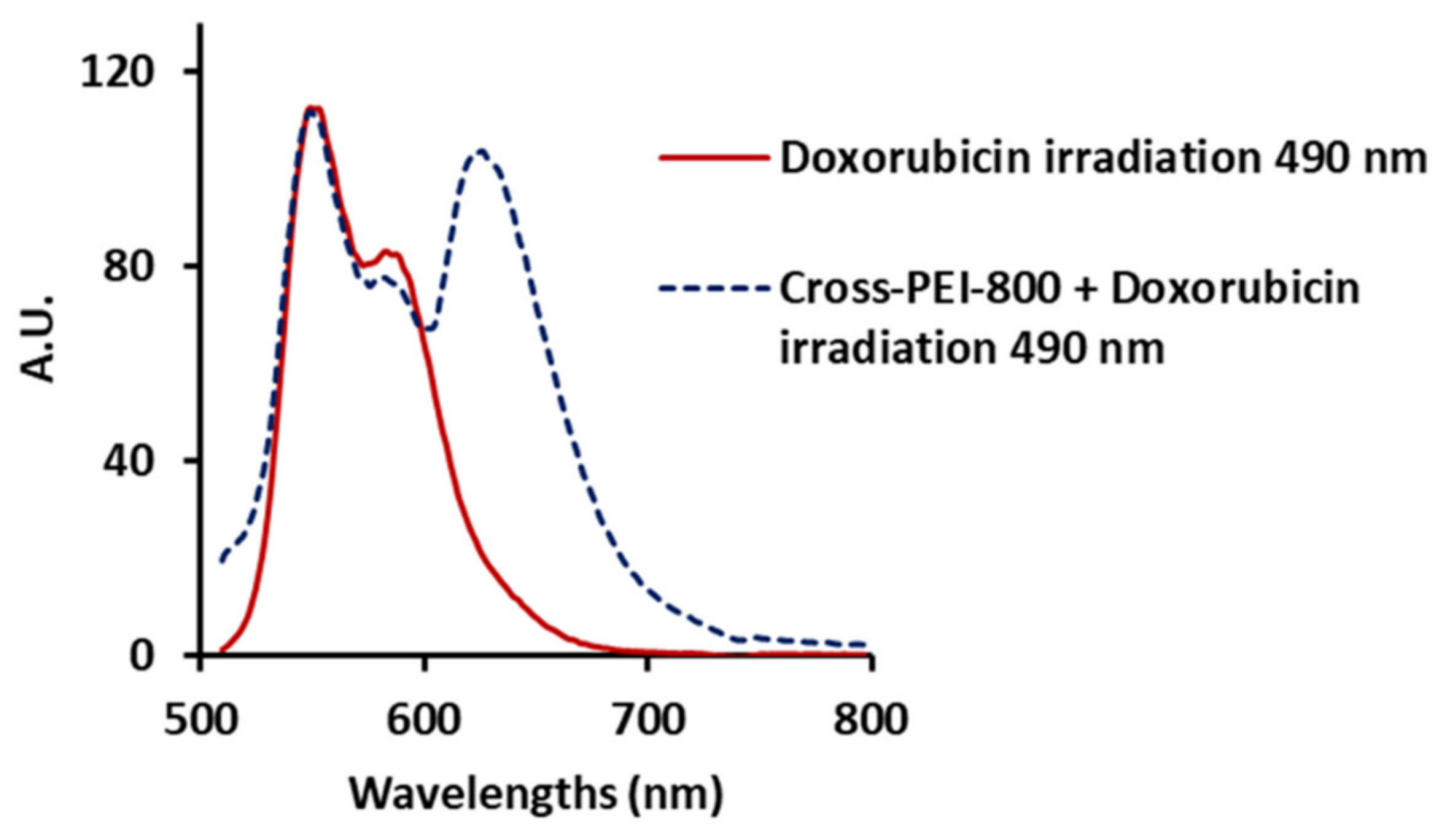
Fluorescence spectra upon irradiation at 490 nm of DOX 5.7 × 10^−6^ M (red line) and DOX 5.7 × 10^−6^ M mixed with **Cross-PEI-800** 1.5 × 10^−4^ M (blue dashed line).

The UV-Vis spectrum of DOX after adding **Cross-PEI-800** displayed absorption broadening with a lowered absorbance (abs) maximum, while after adding **PEI-800**, it showed a red shift of absorption (Figure S10 in the Supplementary Materials). The UV titration analysis revealed that the coordination between **Cross-PEI-800** and DOX followed stoichiometry 1:1, and the equilibrium constant of the complex was 10^7^ (Figure S11 in the Supplementary Materials). As shown in Figure 5 (red line), the irradiation of the DOX solution at 490 nm exhibited a fluorescence maximum at 550 nm, whereas there was no absorption at 490 nm or emission at 550 nm for **Cross-PEI-800** (Figure 3). The addition of **Cross-PEI-800** and the irradiation at the same frequency (490 nm) showed two fluorescence maxima at 550 and 630 nm (blue dashed line, Figure 5). The irradiation at the absorption maxima of the cross-linked polymer (286 and 328 nm) produced only the fluorescence maxima of the polymer (Figure S12 in the Supplementary Materials).

Based on the fluorescence profile of **Cross-PEI-800** incorporated with DOX, the excited electrons of DOX are proposed to migrate to the LUMO orbital of **Cross-PEI-800** and subsequently generate the emission maximum of the cross-linked polymer at 630 nm. Accordingly, this provides further evidence of the molecular interactions between DOX and **Cross-PEI-800**.

#### 3.2.2. Gatifloxacin (Hydrophilic Drug)

**Figure 6:**
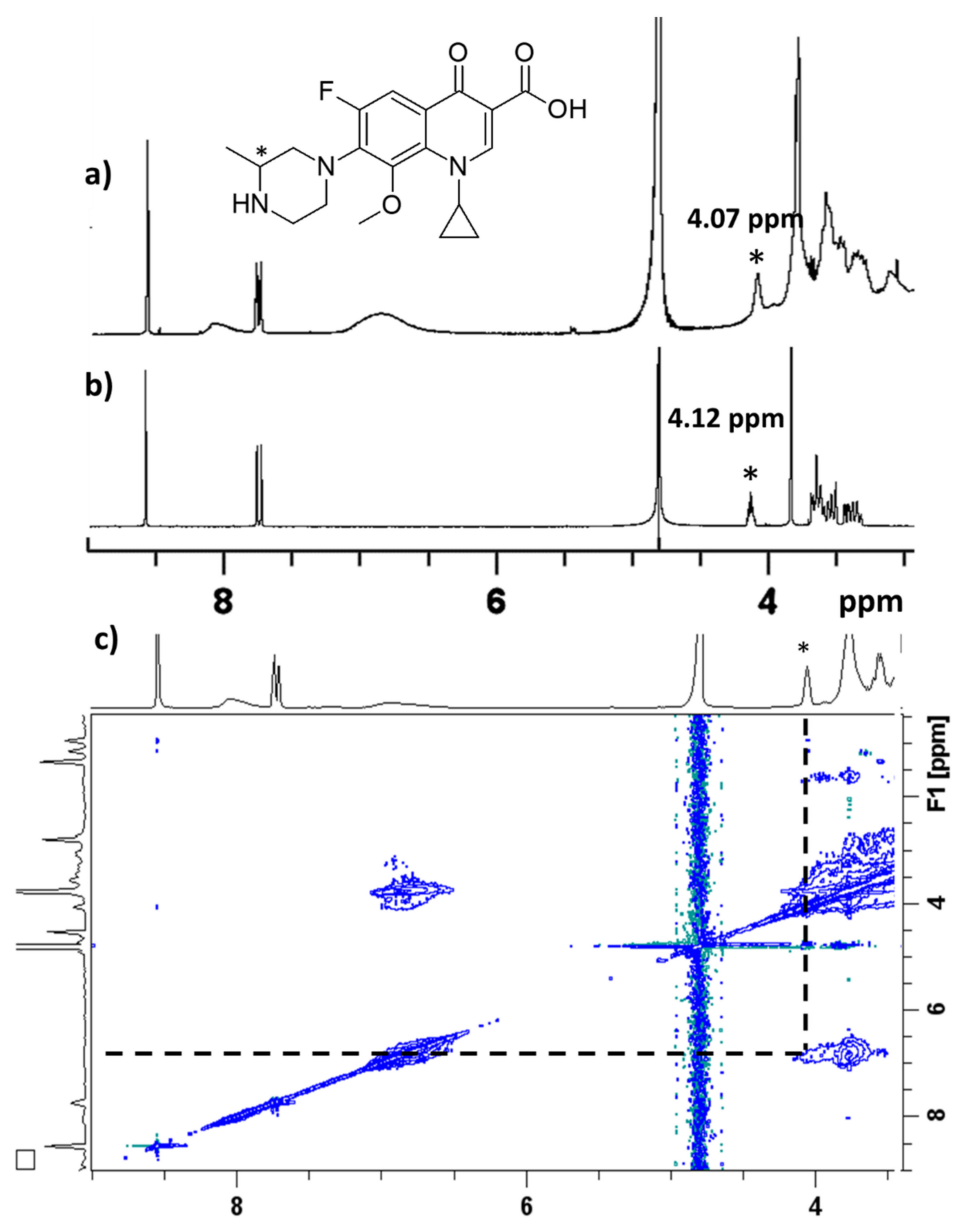
(**a**) ^1^H NMR spectrum, D_2_O, 3–9 ppm, of gatifloxacine mixed with **Cross-PEI-800**, (**b**) ^1^H NMR spectrum D_2_O, 3–9 ppm, of gatifloxacin, (**c**) NOESY spectrum in D_2_O, 2–9 ppm of gatifloxacine mixed with **Cross-PEI-800**. The 1HNMR signal marked with * refers to the hydrogen linked to the tertiary carbon of gatifloxacine piperazine group.

The NMR assignment of gatifloxacin in D_2_O was carried out as reported in the literature [70]. The proton signal of –CH- linked to the secondary amine of gatifloxacine was shifted from 4.12 ppm to 4.07 ppm after the addition of **Cross-PEI-800** (Figure 6a,b, full spectra shown in Figures S13–S15 in the Supplementary Materials). At the same time, the proton signals of **Cross-PEI-800** were overlapped with the –CH_2_- signals of the piperazine moiety of gatifloxacine. In the NOESY spectrum, the signal at 4.07 ppm was found to interact with the aromatic signals of **Cross-PEI-800** (Figure 6c, full spectrum shown in Figure S16 in the Supplementary Materials). NMR data clearly indicated the interaction of the gatifloxacin’s piperazine ring with the CTV moiety of **Cross-PEI-800**.

After mixing gatifloxacin with the cross-linked polymer, the UV-Vis spectrum of gatifloxacin remained unchanged (Figure S17 in the Supplementary Materials). Upon irradiation at the absorption maxima (232, 286 and 330 nm), gatifloxacin produced a blue emission at 430 nm. In the presence of **Cross-PEI-800**, this emission was also observed upon irradiation at 286 and 330 nm. However, irradiation at 232 nm exhibited the emission of the cross-linked polymer (Figure S18 in the Supplementary Materials). According to the previous studies, the absorption of quinolone derivatives at 230 nm is attributed to the fluorophenyl piperazine unit [71]. The irradiation at 230 nm in the presence of the polymer illustrated the disappearance of the drug emission, showing the interaction of fluorophenyl piperazine moiety with **Cross-PEI-800** (Figure S18 in the Supplementary Materials). Accordingly, NMR and UV analyses conclude that **Cross-PEI-800**, with gatifloxacin, exhibits hydrophobic interaction between the piperazine substituent and the proximity of the armed CTV.

#### 3.2.3. Sinomenine (Hydrophilic Drug)

**Figure 7:**
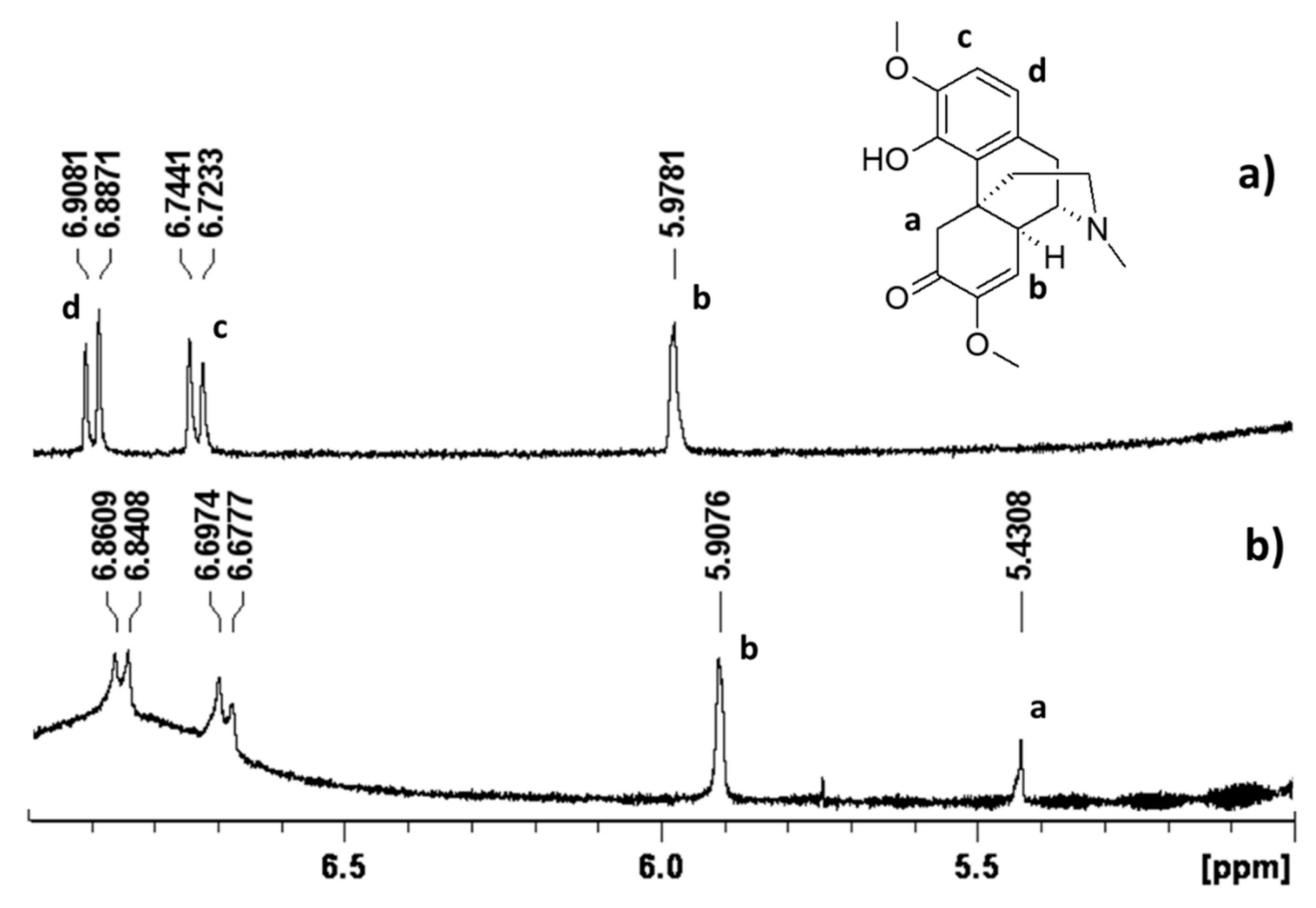
**^1^**H-NMR spectrum in D_2_O, 5–7 ppm of (**a**) pure sinomenine and (**b**) sinomenine mixed with **Cross-PEI-800**. The full-range ^1^H-NMR and NOESY spectra were provided in the Supplementary Materials.

After the addition of **Cross-PEI-800** to sinomenine in the D_2_O solution, the aromatic proton signals of sinomenine were shifted upfield, and a new signal at 5.43 ppm was observed. In accordance with the ^1^H NMR assignment of sinomenine in DMSO-d_6_ [72], the new peak corresponds to the proton at position a (Figure 7, full spectra shown in Figures S19–S21 in the Supplementary Materials) that was absent in the pure drug due to deuterium exchange. Therefore, NMR data demonstrated that the cross-linked polymer partially shielded sinomenine from the interactions with the solvent. Moreover, the upfield shift of the aromatic proton signals proved the existence of π−π interactions between sinomenine and **Cross-PEI-800**. The shifting of b signal and its NOE correlations with the –OMe signal of polymer indicated the hydrophobic and p-p interaction between sinomenine and armed CTV unit.

Besides, the UV-Vis spectrum of sinomenine showed an absorption band at 264 nm, and its intensity was enhanced after treatment with **Cross-PEI-800** (Figure S22a). Similar to other drug molecules, the emission spectra can also identify the associated intermolecular interactions. The irradiation of sinomenine at 264 nm (abs maximum) showed no emission. Upon treatment with **Cross-PEI-800**, the emission maxima of the cross-linked polymer (320 and 630 nm upon irradiation at 286 nm) along with an additional maximum at 430 nm were found (Figure S22b). Irradiations of the mixture at 286 and 324 nm generated the same absorption maxima as pure **Cross-PEI-800** (Figure S22c,d).

#### 3.2.4. Camptothecin (Hydrophobic Drug)

**Figure 8:**
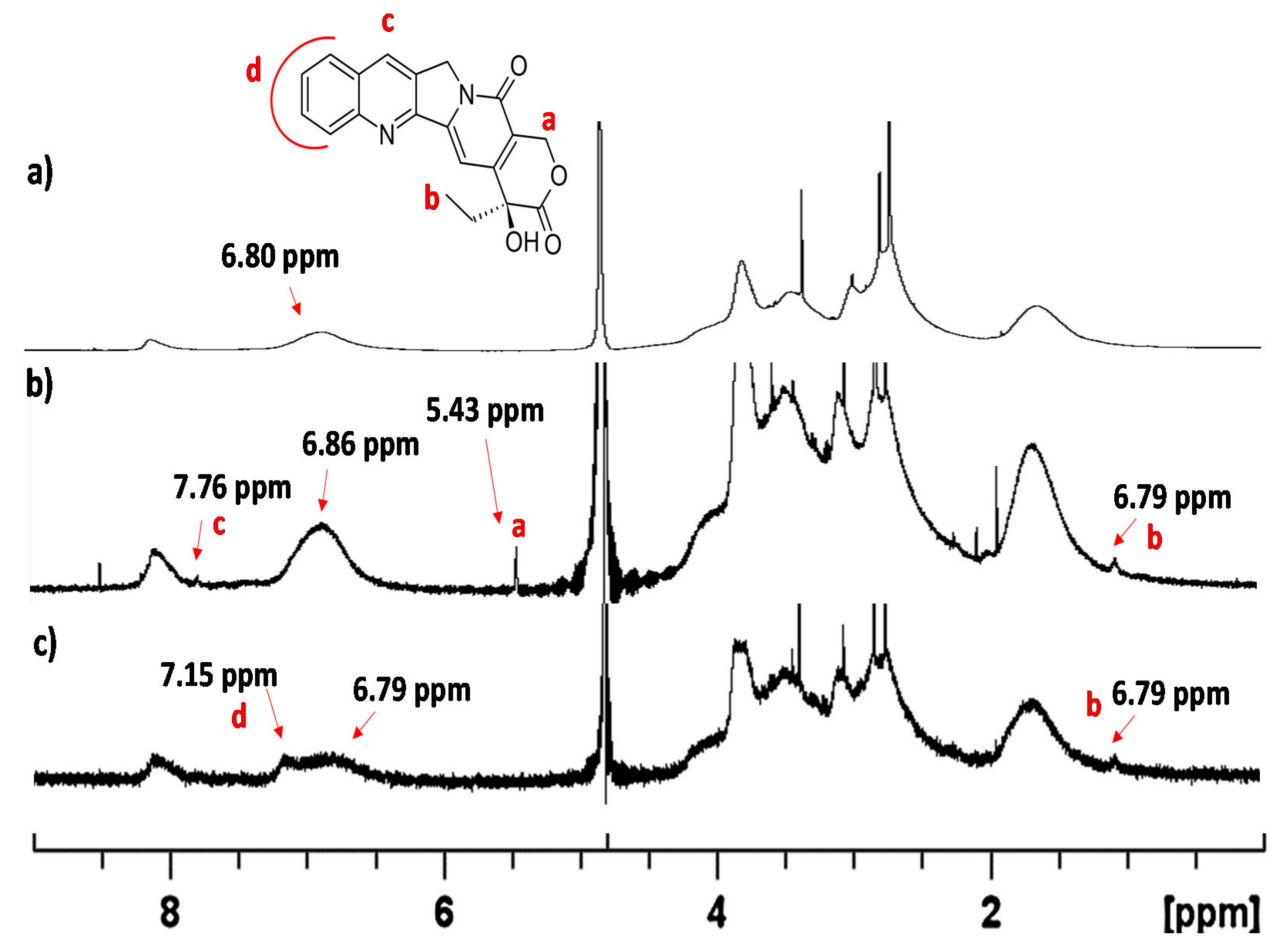
(**a**) ^1^H-NMR in D_2_O spectrum of **Cross-PEI-800** range 0–9 ppm, (**b**) ^1^H-NMR spectrum in D_2_O of camptothecin (5 mg) in the presence of **Cross-PEI-800** (5 mg), range 0–9 ppm, (**c**) ^1^H-NMR spectrum in D_2_O of camptothecin (7 mg) in the presence of **Cross-PEI-800** (2 mg), range 0–9 ppm.

Camptothecin in D_2_O alone did not exhibit any NMR signals. In the presence of a large amount of **Cross-PEI-800***,* some proton peaks of camptothecin were found and assigned as **a**, **b** and **c** (Figure 8b) based on the reported NMR assignments of camptothecin in DMSO-d_6_, Trifluoroacetic acid-d and modified camptothecin inD_2_O solutions [73,74,75]. By reducing the amount of **Cross-PEI-800**, the aromatic proton signals of camptothecin became overlapped with those of the cross-linked polymer, and they were assigned as **d** in Figure 8c (enlarged spectra shown in Figures S23–S25).

Regarding the NOESY spectrum of camptothecin in the presence of the cross-linked polymer, the aromatic signal at 7.15 ppm interacted with the –OMe signals at 3.82 ppm of **Cross-PEI-800** (Figure S26 in the Supplementary Materials). On the other hand, camptothecin in H_2_O exhibited weak UV absorption, which increased the intensity after the addition of **Cross-PEI-800** (Figure S27 in the Supplementary Materials). When switching to **PEI-800**, the intensity also increased with a red shift. These results further prove the drug’s tendency to coordinate the hydrophobic armed CTV unit of **Cross-PEI-800**. Pure camptothecin upon irradiation generated emission at 430 nm that was also visible in the presence of **Cross-PEI-800** (Figure S28 in the Supplementary Materials). The titration experiments of the drug with the polymer revealed that two units of armed CTV were involved in the coordination with camptothecin (Figure S29 in the Supplementary Materials).

#### 3.2.5. Celastrol (Hydrophobic Drug)

In the D_2_O solution, no proton signal of pure celastrol was observed, whereas only the polymer’s signals were found after adding **Cross-PEI-800,** owing to the overlapping by intense proton signals of the polymer (Figures S30–32 in the Supplementary Materials). In the solution with fewer polymers, there was a very weak additional signal at 7.15 ppm, similar to the case of camptothecin. This was due to the poor water solubility of the drug. This signal was more distinguishable in the NOESY spectrum (Figure S33 in the Supplementary Materials).

Other studies have reported that the UV-Visible spectroscopy method was employed for the reversibility studies of celastrol [76]. The spectra of celastrol in phosphate-buffered saline (pH 7.4) were reported to exhibit a peak at 440 nm [77]. Further studies revealed that quinone methide rapidly reacted with thiol groups under physiological conditions and subsequently caused a decrease in the UV-Visible absorption, indicating the formation of covalent adducts.

**Figure 9:**
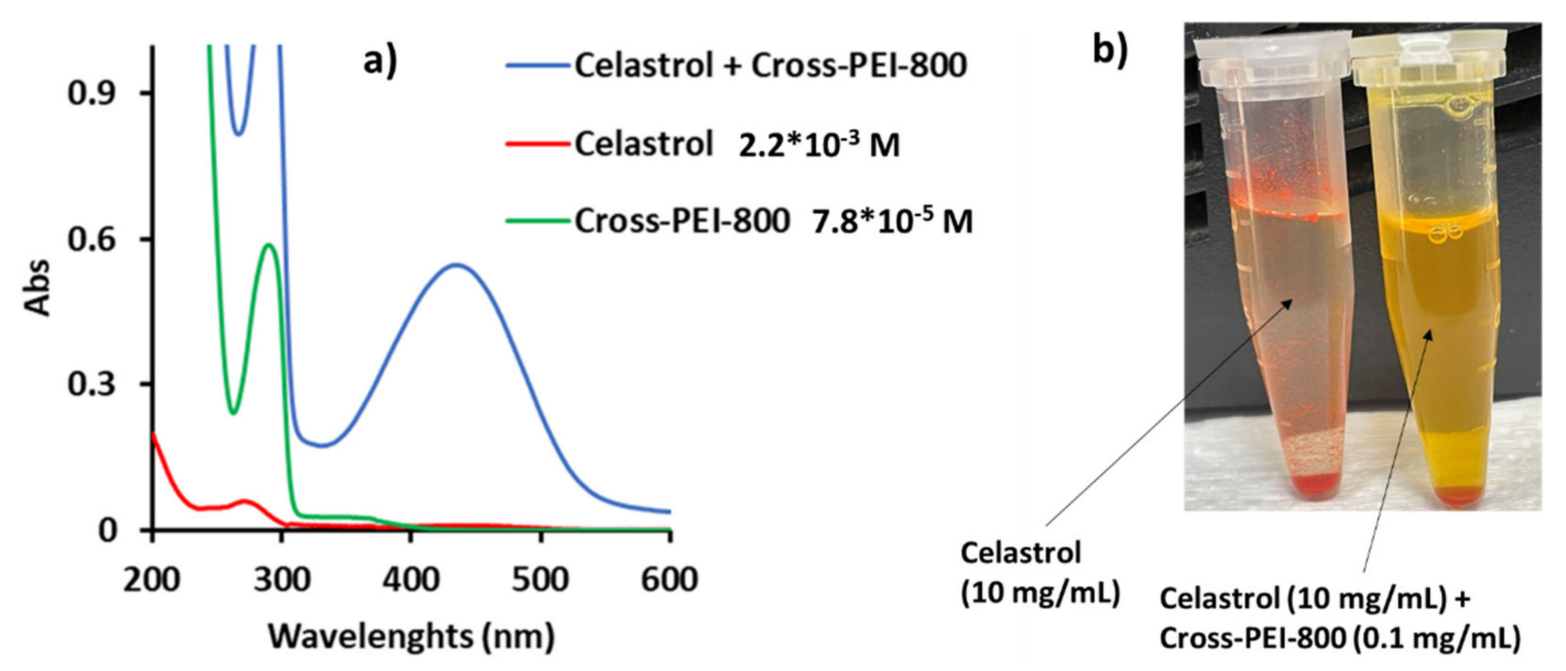
(**a**) UV-Vis spectrum of pure celastrol, pure **Cross-PEI-800**, celastrol mixed with **Cross-PEI-800** (1% of cross-linked polymer), (**b**) Chromatic solution variation after addition of polymer to celastrol.

In our experiments, the UV-Vis absorption spectrum of celastrol in water only displayed a weak absorption at 280 nm (Figure 9a). The addition of 1% (in weight) of **Cross-PEI-800** formed a new absorption band at 435 nm and resulted in a color change in the solution (Figure 9a,b, as detailed by the UV-Vis analysis shown in Figure S34 in the Supplementary Materials).

In addition, the irradiation of the mixture at 435 nm displayed the emission peaks of **Cross-PEI-800**. In other words, the new absorption at 435 nm cannot be ascribed to a modification of celastrol but to a complex formation between the drug and the cross-linked polymer (Figure S35 in the Supplementary Materials).

Subsequently, titration experiments found that the coordination between celastrol and **Cross-PEI-800** was stoichiometric, in a ratio of 1:1 (Figure S36 in the Supplementary Materials). The drug solubility in water increased 100 times after adding 1% of **Cross-PEI-800**; more importantly, the solubility further increased after 10 days (Figure S37 in the Supplementary Materials). Furthermore, the absorption band of the mixture was shifted from 435 to 360 nm after 10 days, as shown in the UV-Vis spectra (Figure S38 in the Supplementary Materials). This absorption change was postulated to involve a change of interactions between celastrol and **Cross-PEI-800**. The absorption of the drug-polymer mixture at 435 nm (blue line, Figure 9) was similar to that of celastrol in phosphate buffer at pH 7. This suggested a similar interaction due to the buffering power of the polyethylenimine group. Meanwhile, the blue shift of the absorption band after 10 days is attributed to a hydrophobic or π−π stacking interaction.

### 3.3. Guest Molecule Releasing Preliminary Analysis

To understand the capability of releasing the guest molecule from the hosting system, DOX (hydrophilic) and celastrol (hydrophobic), which interacted with **Cross-PEI-800**, were subjected to a pH change using NH_4_Cl. With the addition of NH_4_Cl to the solutions, the pH of the solution was maintained at 7, and the recovery of DOX absorption was found except for celastrol (Figure S39 in the Supplementary Materials). The results suggested that the DOX can be easily released from **Cross-PEI-800** under an acidic intracellular environment. In the case of celastrol, the releasing process requires different conditions, for example, the addition of a competitive guest compound.

## 4. Discussion

The ^1^H-NMR spectrum of **Cross-PEI-800** in D_2_O revealed that the PEI moiety assisted in the water dissolution of the hydrophobic armed CTV functional group. In the ^1^H-NMR spectrum of **Cross-PEI-800** (Figure 2), the assignment of all proton signals of armed CTV unit can be achieved by comparison with the spectrum of **Armed-CTV** in CDCl_3_ (Figure S3 in the Supplementary Materials). The same information was obtained by comparing the UV spectra of **Armed-CTV** (spectrum in MeOH, Figure S40 in the Supplementary Materials) with **Cross-PEI-800** (Figure 3). The two compounds displayed the same absorption band at ~290 nm with similar coefficients. The irradiation at this frequency exhibited emission (dashed line, Figure 4c), and this indicated that the CTV unit generated the emission.

The ^1^H-NMR and NOESY spectra showed that the aromatic groups of DOX, sinomenine and camptothecin, interacted with the armed CTV unit, respectively. On the contrary, the piperazine group of gatifloxacin was involved in the major interaction with the cross-linked polymer. Furthermore, the interaction of celastrol was detectable from UV-Vis spectra, where the coordination involves the amino and CTV groups of **Cross-PEI-800** and the heteroatoms of the drug.

The emission spectra analysis further proves the drug-CTV unit interaction. Irradiation of the solution mixtures at the drugs’ absorption maxima, the photoluminescence (PL) spectra generated the emission of the cross-linked polymer upon irradiation at 290 nm.

Finally, the addition of NH_4_Cl to the DOX-**Cross-PEI-800** conjugate induced the recovery of the drug’s UV-absorption, owing to the detachment of the drug from the cross-linked polymer by acidic proton. This shows that **Cross-PEI-800** is a viable and potential platform for drug delivery.

## 5. Conclusions

A full-organic, water-soluble material, **Cross-PEI-800**, was prepared by combining two molecular units (CTV and **PEI-800**) often used in supramolecular chemistry that exhibit different coordinating features. CTV is a concave macrocycle that interacts with guest molecules through Van der Waals, π-π stacking and hydrophobic interactions. **PEI-800** is a polyethylenimine that is highly soluble in water and coordinates other molecules through hydrogen bonding or electrostatic interactions. **Cross-PEI-800** consists of a water-soluble gel that contains hydrophobic holes with a high coordinating power towards organic molecules. This material has been demonstrated to encapsulate molecules with different characteristics via various interaction modalities.

Owing to the water solubility and optical properties of the prepared coordinating systems, various spectroscopic methods were employed to examine the host–guest complexes in water under normal conditions. Of note, other research groups also reported coordinating materials, including synthetic organic macrocycles that coordinate drugs in organic solvents and subcritical water [78,79], and the assembly of an organic macrocycle and inorganic nanoparticle that coordinates drugs in water [80].

The synthesized system was found to coordinate both hydrophilic and hydrophobic molecules. Both CTV and **PEI-800** moieties were involved in all drugs’ coordination. The results show that the armed CTV unit works as a hole trap for small aromatic organic molecules. Only celastrol exhibited different coordination induced by the buffering power of the polyethyleneimine unit. The main feature of **Cross-PEI-800** consists of its complexity or multifunctionality, which contributes to various physical properties and coordinating modalities.

In this work, the molecular structure and the coordinating capability of **Cross-PEI-800** were examined. **Cross-PEI-800** not only showed its capability to coordinate the drugs in water but also exhibited relatively low cytotoxicity. Two drug molecules, DOX and celastrol, were selected for the preliminary analysis of the drug-releasing capability of **Cross-PEI-800** using ammonium chloride. The material has shown its capability to slowly release DOX under a simulated intracellular acidic environment, possibly due to the contribution of H^+^ ions to the separation of the guest–host complex.

The presented data demonstrate that the system, along with some modifications, merits further studies about the drug-delivery capability. An in-depth study on the delivery selectivity of **Cross-PEI-800** would be needed. The presented study could be of interest to medical applications. Due to the capability of **PEI-800** in coordinating nucleic acids, the prepared material **Cross-PEI-800** could find application as a gene-drug co-delivery system.

## Figures and Tables

**Table 1:** The spectroscopic and biological properties of **Cross-PEI-800**.

**UV-Vis Frequency Maxima**	λ_1_ = 286 nm, λ_2_ = 328 nm
**Emission frequency maxima (λ irradiation)**	330 nm (λ_1_), 450 nm (λ_2_), 630 nm (λ_1_)
^1^ **H NMR chemical shifts**	8.00 ppm, 6.82 ppm, 1–4 ppm
**Cytotoxicity (μg/mL)**	
A549 cell line	>500
LO2 cell line	59.314
HEK293 cell line	321.59

## Data Availability

All data and analyses are available in the manuscript and in the Supplementary Materials.

## Supplementary Materials

The following are available online at https://www.mdpi.com/article/10.3390/polym13234133/s1, Figure S1: ^1^H-NMR spectrum compound 1a. Figure S2: ^13^C-NMR spectrum compound 1a. Figure S3: ^1^H-NMR second reaction. Figure S4: ^13^C-NMR second reaction. Figure S5: ^1^H-NMR spectrum CossPEI-800, Evaluation of monomer weight from the integration of the NMR signals. Figure S6: ^1^H-NMR spectrum in D_2_O of Cross-PEI-800 mixed with doxorubicin. Figure S7: ^1^H-NMR spectrum in D2O of Cross-PEI-800 mixed with doxorubicin after 1 week. Figure S8: ^1^H-NMR spectrum in D2O of doxorubicin with peak assignment. Figure S9: NOESY spectrum of doxorubicin mixed with Cross-PEI-800. Figure S10: UV-Vis spectra of doxorubicin (DOX), PEI-800, Cross-PEI-800 in pure form and in mixed solutions. Figure S11: Titration of doxorubicin (DOX) with Cross-PEI-800. Figure S12: Fluorescence spectra of the doxorubicin mixed with Cross-PEI 800. Figure S13: ^1^H-NMR spectrum of Gatifloxacin. Figure S14: ^1^H-NMR spectrum of Cross-PEI-800 mixed with Gatifloxacin (5 mg + 5 mg). Figure S15: Comparation between ^1^H-NMR spectra of gatifloxacine, Cross-PEI-800, gatifloxacine + Cross-PEI-800. Figure S16: NOESY spectrum of Gatifloxacin mixed with Cross-PEI-800. Figure S17: UV-Vis spectra of Gatifloxacin with and without crosslinked polymer. Figure S18: Fluorescence spectra of gatifloxacine and gatifloxacine mixed with Cross-PEI-800. Figure S19: ^1^H-NMR spectrum in D2O of sinomenine. Figure S20: ^1^H-NMR spectrum of sinomenine in the presence of Cross-PEI-800 (5mg + 5mg). Figure S21: NOESY spectrum of sinomenine in the presence of Cross-PEI-800 (5mg + 5mg). Figure S22: UV-Vis and emission spectra of SIN pure and in the presence of Cross-PEI-800. Figure S23: ^1^H-NMR spectrum of Camptothecine in the presence of Cross-PEI-800. Figure S24: ^1^H-NMR spectrum of Camptothecine in the presence of Cross-PEI-800 enlarged. Figure S25: ^1^H-NMR spectrum of Camptothecine in the presence of Cross-PEI-800. Figure S26: NOESY spectrum of Camptothecine in the presence of Cross-PEI-800. Figure S27: UV-Vis spectra of Camptothecin pure and in the presence of Cross-PEI-800, PEI-800. Figure S28: Emission spectra of Camptothecin. Figure S29: Titration of Camptothecin with Cross-PEI-800. Figure S30: ^1^H-NMR spectra of Cross-PEI-800 and celastrol in the presence of Cross-PEI-800. Figure S31: ^1^H-NMR spectrum of celastrol in the presence of Cross-PEI-800. Figure S32: ^1^H-NMR of celastrol in the presence of Cross-PEI-800 (second dosage of polymer). Figure S33: NOESY spectrum of celastrol in the presence of Cross-PEI-800. Figure S34: UV-Vis spectra of celastrol solution, pure and in the presence of Polymer Cross-PEI-800. Figure S35: Emission spectra of celastrol pure and in the presence of Cross-PEI-800, PEI-800. Figure S36: UV-Vis titration of celastrol with Cross-PEI-800, PEI-800. Figure S37: Water solubility measures of celastrol pure and in the presence of Cross-PEI-800. Figure S38: UV-Vis spectra of celastrol mixed with Cross-PEI-800 after 10 days. Figure S39: UV-Vis spectra of doxorubicin and celastrol in the presence of Cross-PEI-800 and addition of NH_4_Cl. Figure S40: UV-Vis spectrum of Armed-CTV in methanol. Table S1: Cytotoxicity of polymer Cross-PEI-800 against normal and cancer cell lines.
