# Supplementary material for: Synthesis and Coordination Properties of a Water-Soluble Material by Cross-Linking Low Molecular Weight Polyethyleneimine with Armed Cyclotriveratrilene
Source: Polymers (Basel). 2021 Nov 26;13(23):4133. doi: 10.3390/polym13234133 (PMC8659696; doi:10.3390/polym13234133)
Supplement: Supplementary file 1 [file polymers-13-04133-s001.zip › polymers-1449844-supplementary.pdf]

Synthesis and coordination properties of a water-soluble material by cross-linking low molecular weight polyethyleneimine with armed cyclotriveratrilene

Yoke Mooi Ng[1,†], Paolo Coghi[2,†], Jerome P. L. Ng [3†], Fayaz Ali [4], Vincent Kam Wai Wong[-4,3] and Carmine Coluccini[1*]

[1] Institute of New Drug Development, College of Medicine , China Medical University, No.91 Hsueh-Shih Road, Taichung 40402, Taiwan; yumei.eu@gmail.com (Y. M. N)
[2] School of Pharmacy, Macau University of Science and Technology, 999078 Macau, China; coghips@must,edu.mo (P.C.)
[3] Neher's Biophysics Laboratory for Innovative Drug Discovery, State Key Laboratory of Quality Research in Chinese Medicine, Macau University of Science and Technology, 999078 Macau, China; plng@must.edu.mo (J.P.L.N.); bowaiwong@gmail.com (V.K.W.W.)
[4] Department chemistry, Abbottabad University of Science and Technology, 22010 Abbottabad, Pakistan; fayazalisabir@gmail.com[4]
Neher's Biophysics Laboratory for Innovative Drug Discovery, State Key Laboratory of Quality Research in Chinese Medicine, Macau University of Science and Technology, Macau, China; bowaiwong@gmail.com (V.K.W.W.);
* Correspondence: carmine.coluccini@mail.cmu.edu.tw (C.C.); Tel.: +886-422053366 #8200;. † These authors contributed equally

Contents

Figure S1. ^{1}H-NMR spectrum compound 1a 3

Figure S2. ^{13}C-NMR spectrum compound 1a. 3

Figure S3. ^{1}H-NMR second reaction 4

Figure S4. ^{13}C-NMR second reaction 4

Figure S5. ^{1}H-NMR spectrum CossPEI-800. 5

Appendix of Figure 5. ***Evaluation of monomer weight from the integration of the NMR signals... 5***

Figure S6. ^{1}H-NMR spectrum in D_2O of Cross-PEI-800 mixed with Doxorubicin. 6

Figure S7. ^{1}H-NMR spectrum in D2O of Cross-PEI-800 mixed with Doxorubicin after 1 week 6

Figure S8. ^{1}H-NMR spectrum in D2O of Doxorubicin with peak assignament. 7

Figure S9. NOESY spectrum of doxorubicin mixed with Cross-PEI-800. The dash red line marked with a refers to the interactions of aromatic signals with –OMe, -OCH2- signals 7

Figure S10. UV-Vis spectra of Doxorubicin (DOX), PEI-800, Cross-PEI-800 in pure form and in mixed solutions. 8

Figure S11. Titration of Doxorubicin (DOX) with Cross-PEI-800 8

Figure S12. Fluorescence spectra of the Doxorubicin mixed with Cross-PEI 800 9

Figure S13. ^{1}H-NMR spectrum of Gatifloxacin. 10

Figure S14. ^{1}H-NMR spectrum of Cross-PEI-800 mixed with Gatifloxacin (5 mg + 5 mg). 10

Figure S15. Comparation between ^{1}H-NMR spectra of Gatifloxacine, Cross-PEI-800, Gatifloxacine + Cross-PEI-800. 11

Figure S16. NOESY spectrum of Gatifloxacin mixed with Cross-PEI-800 11

Figure S17. UV-Vis spectra of Gatifloxacin with and without crosslinked polymer 12

Figure S18. Fluorescence spectra of Gatifloxacine and Gatifloxacine mixed with Cross-PEI-800 ... 13
Figure S19. ^{1}H-NMR spectrum in D2O of Sinomenine ... 13
Figure S20. ^{1}H-NMR spectrum of Sinomenine in the presence of Cross-PEI-800 (5mg + 5mg). ... 14
Figure S21. NOESY spectrum of Sinomenine in the presence of Cross-PEI-800 (5mg + 5mg). ... 14
Figure S22. UV-vis and emission spectra of SIN pure and in the presence of Cross-PEI-800 ... 15
Figure S23. ^{1}H-NMR spectrum of Camptothecine in the presence of Cross-PEI-800 ... 15
Figure S24. ^{1}H-NMR spectrum of Camptothecine in the presence of Cross-PEI-800 enlarged ... 16
Figure S25. ^{1}H-NMR spectrum of Camptothecine in the presence of Cross-PEI-800 ... 16
Figure S26. NOESY spectrum of Camptothecine in the presence of Cross-PEI-800 ... 17
Figure S27. UV-Vis spectra of Camptothecin pure and in the presence of Cross-PEI-800, PEI-800 17
Figure S28. Emission spectra of Camptothecin ... 18
Figure S29. Titration of Camptothecin with Cross-PEI-800 ... 18
Figure S30. ^{1}H-NMR spectra of Cross-PEI-800 and Celastrol in the presence of Cross-PEI-800 ... 19
Figure S31. ^{1}H-NMR spectrum of Celastrol in the presence of Cross-PEI-800 ... 19
Figure S32. ^{1}H-NMR of Celastrol in the presence of Cross-PEI-800 (second dosage of polymer) . 20
Figure S33. NOESY spectrum of Celastrol in the presence of Cross-PEI-800 ... 20
Figure S34. UV-vis spectra of Celastrol solution, pure and in the presence of Polymer Cross-PEI-800 ... 21
Figure S35. Emission spectra of Celastrol pure and in the presence of Cross-PEI-800, PEI-800 ... 22
Figure S36. UV-Vis titration of Celastrol with Cross-PEI-800, PEI-800 ... 22
Figure S37. Water solubility measures of Celastrol pure and in the presence of Cross-PEI-800 ... 23
Figure S38. UV-Vis spectra of Celastrol mixed with Cross-PEI-800 after 10 days ... 23
Figure S39. UV-Vis spectra of Doxorubicin and Celastrol with Cross-PEI-800 and NH_4Cl ... 24
Figure S40. UV-Vis spectrum of Armed-CTV in Methanol ... 24
Table 1. Cytotoxicity of polymer Cross-PEI-800 against normal and cancer cell lines ...
Abbreviations ... 24

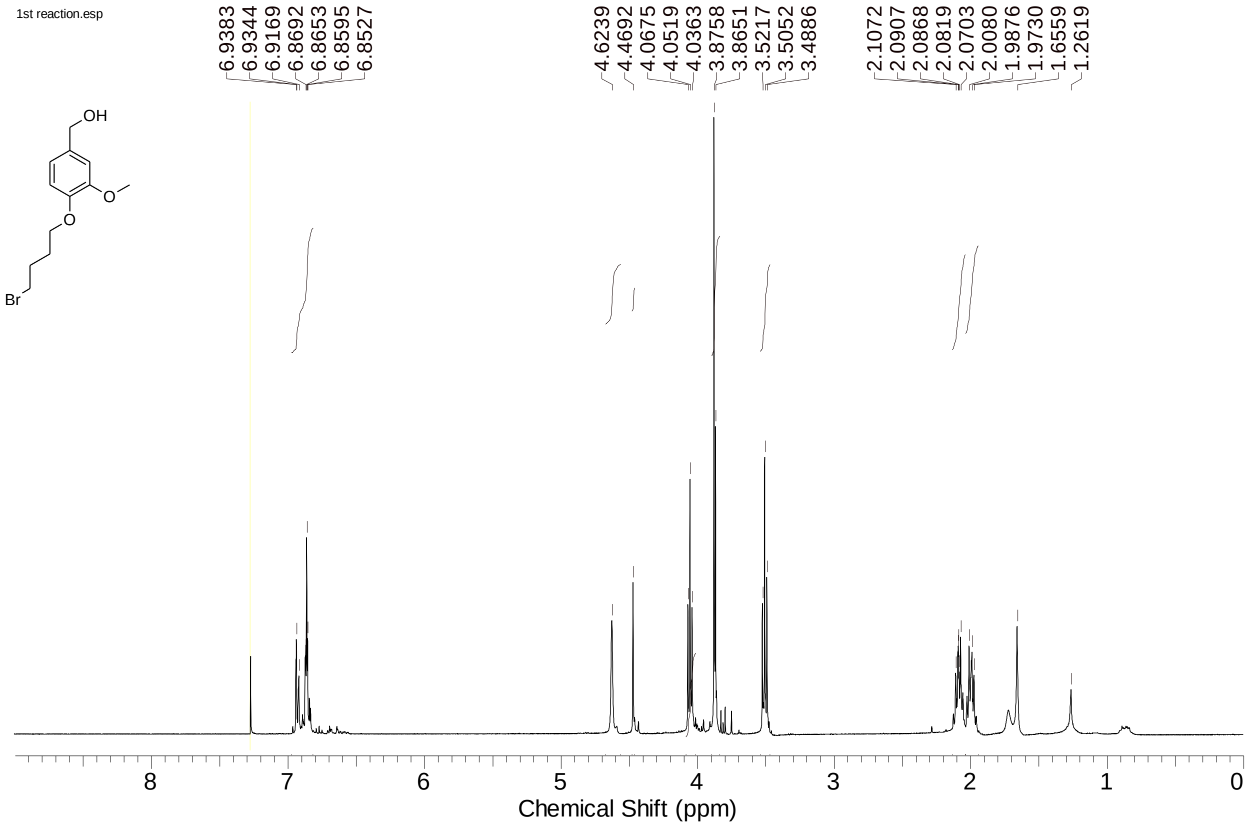

Figure S1. ^{1}H-NMR spectrum compound **1a**.

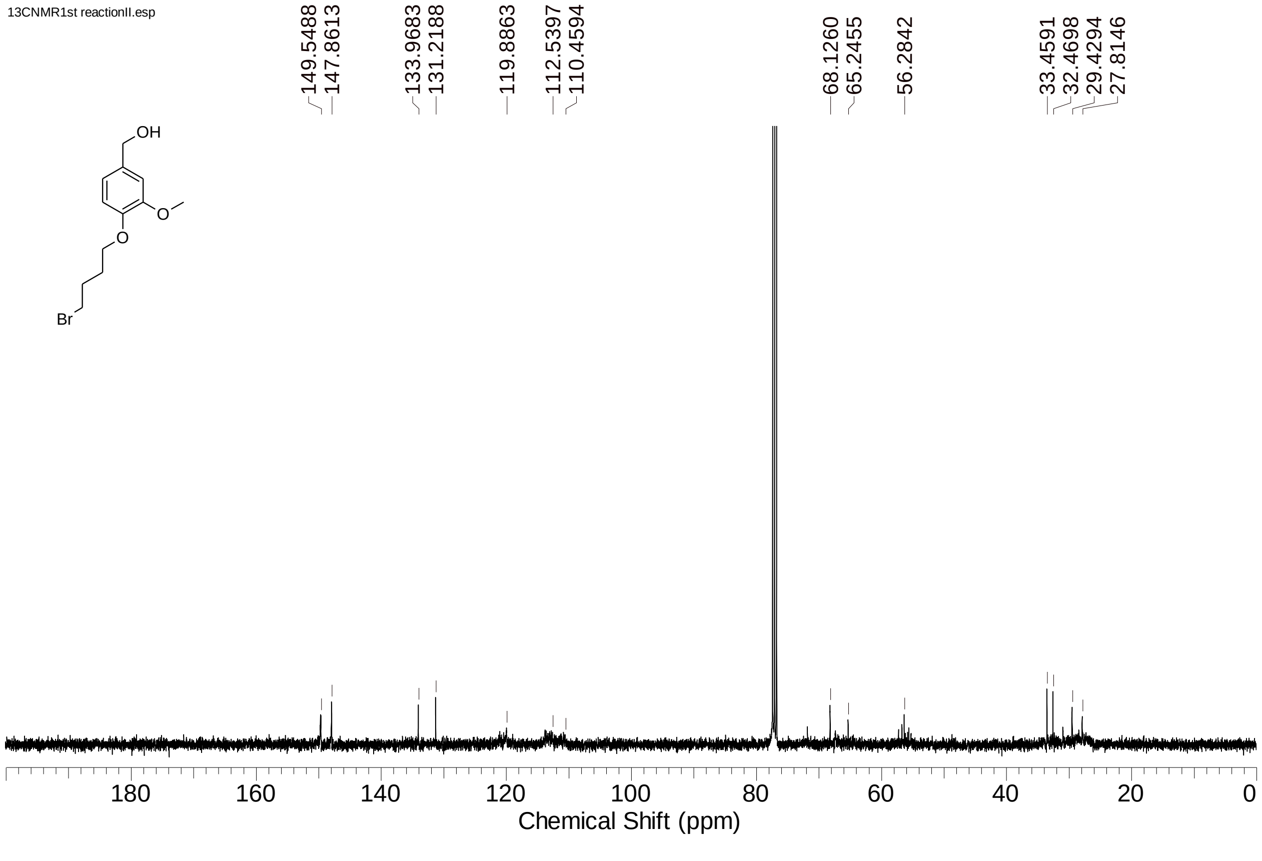

Figure S2. ^{13}C-NMR spectrum compound **1a.**

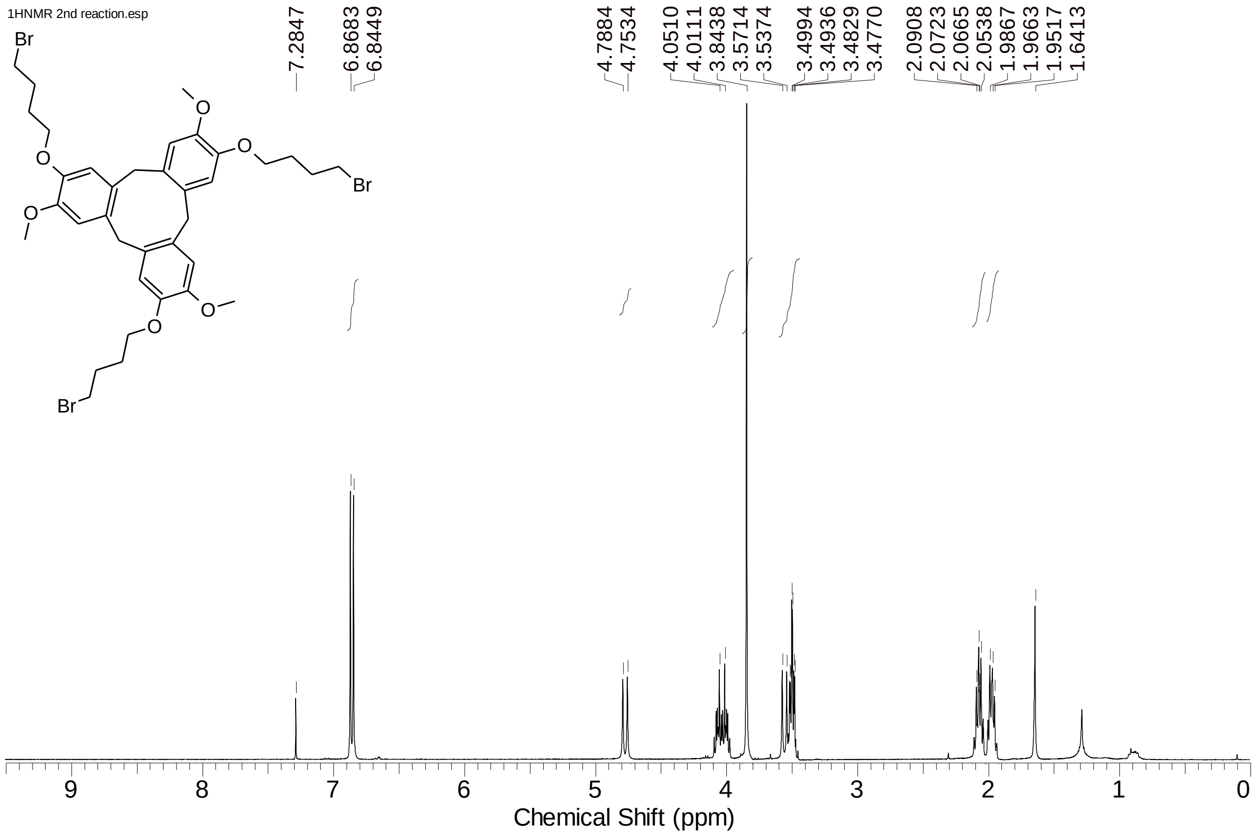

Figure S3. Armed-CTV ¹H-NMR spectrum.

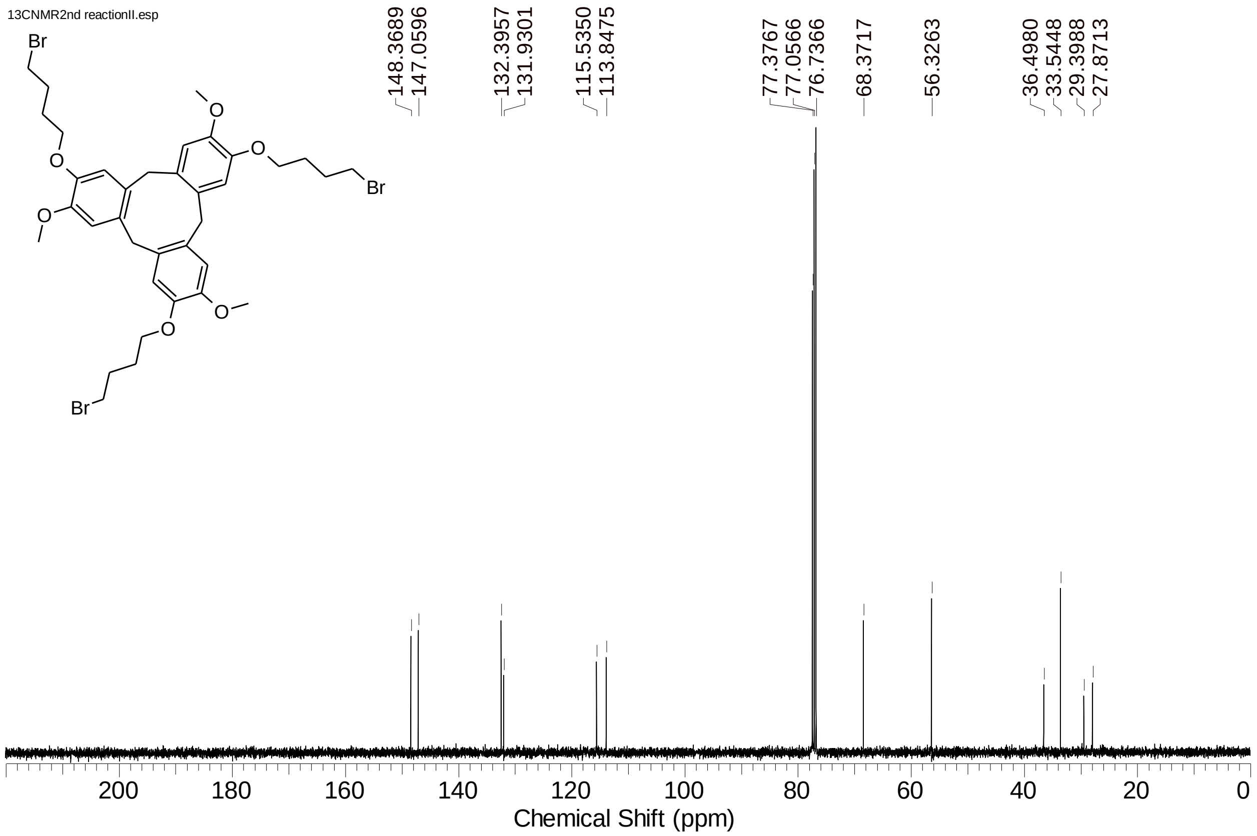

Figure S4. Armed-CTV ¹³C-NMR spectrum.

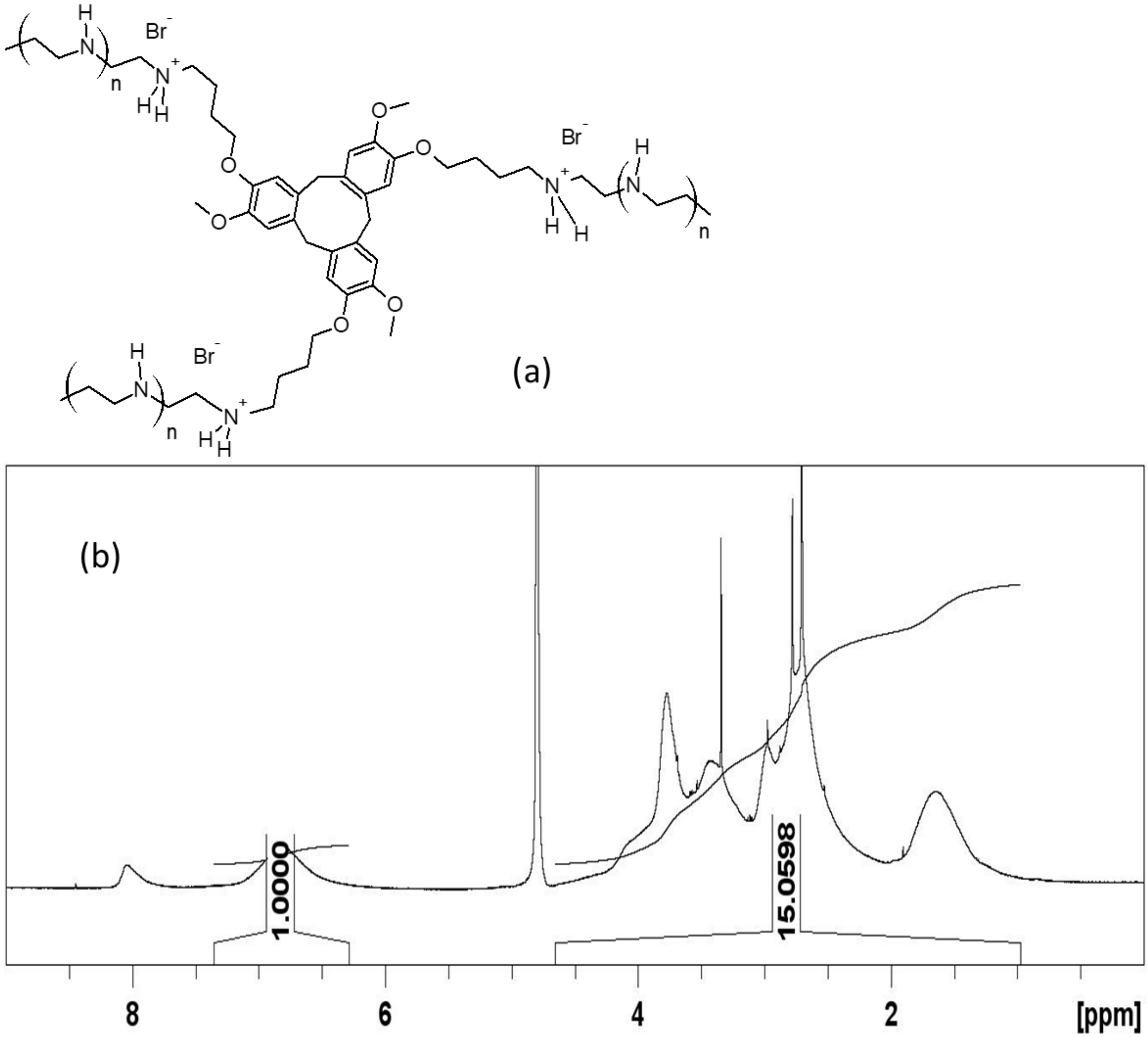

Figure S5. Cross-PEI-800 ^{1}H-NMR spectrum.

Appendix of Figure S5. ***Monomer weight evaluation from the integration of the NMR signals***

According with the structure in figure S5-a the monomer contain 6 aromatic hydrogens, 6 hydrogens linked to the charged nitrogen and 51 + 15n haliphatic and amine hydrogens. According with integration in the figure S5-b the rate between aromatic and no aromatic hydrogens is 1/15 (the signal at 8ppm refers to the hydrogens linked to the charged nitrogen). We can write the following relationship:

$1/15 = 6/(51+15n) \Rightarrow n=2.6$

It means that n is the average of ethylamine groups linked to each alkyl chain of armed CTV unit. If n of figure S5-a is 2.6 and the weight of the fragment –$NHCH_2CH_2$- is 43.07, we can evaluate the molecular weight of the monomer 1277.4 uma.

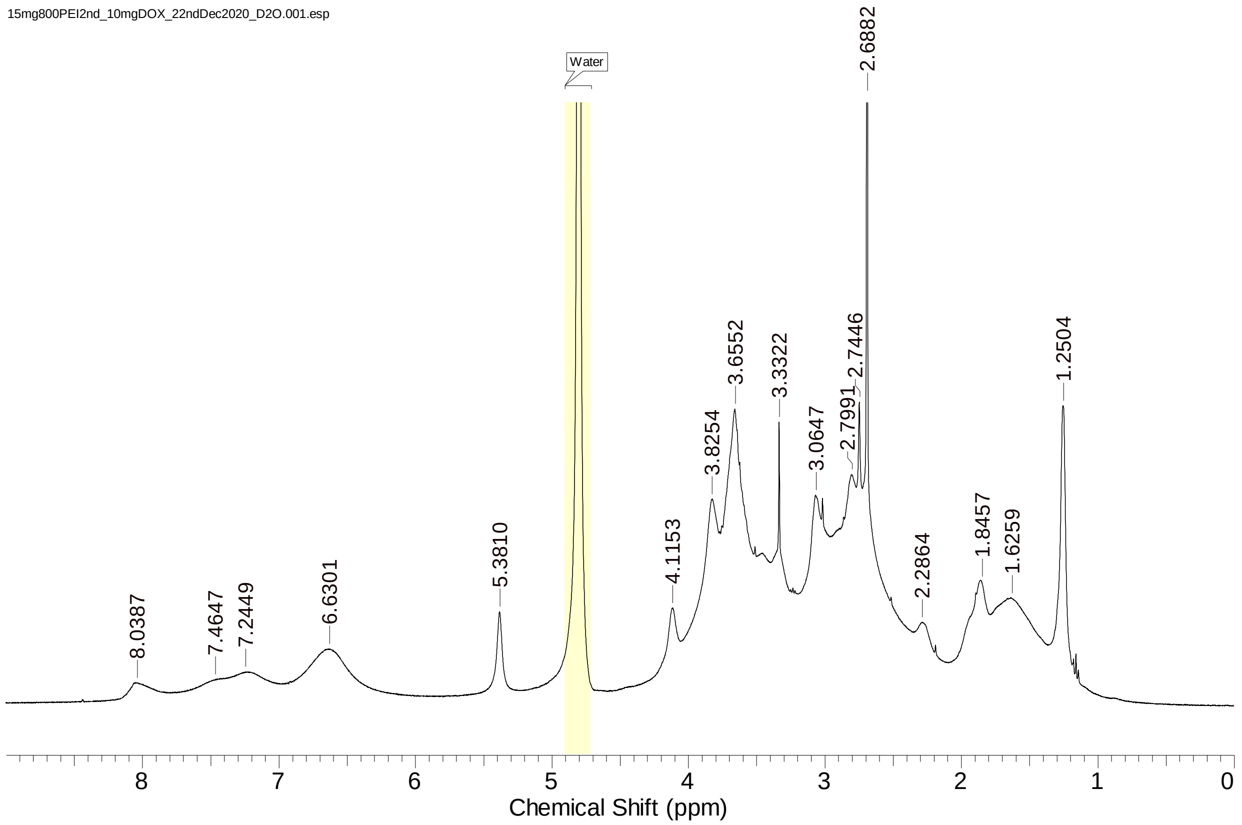

Figure S6. ^{1}H-NMR spectrum in D_2O, 0-9 ppm ranged, of **Cross-PEI-800** mixed with Doxorubicin.

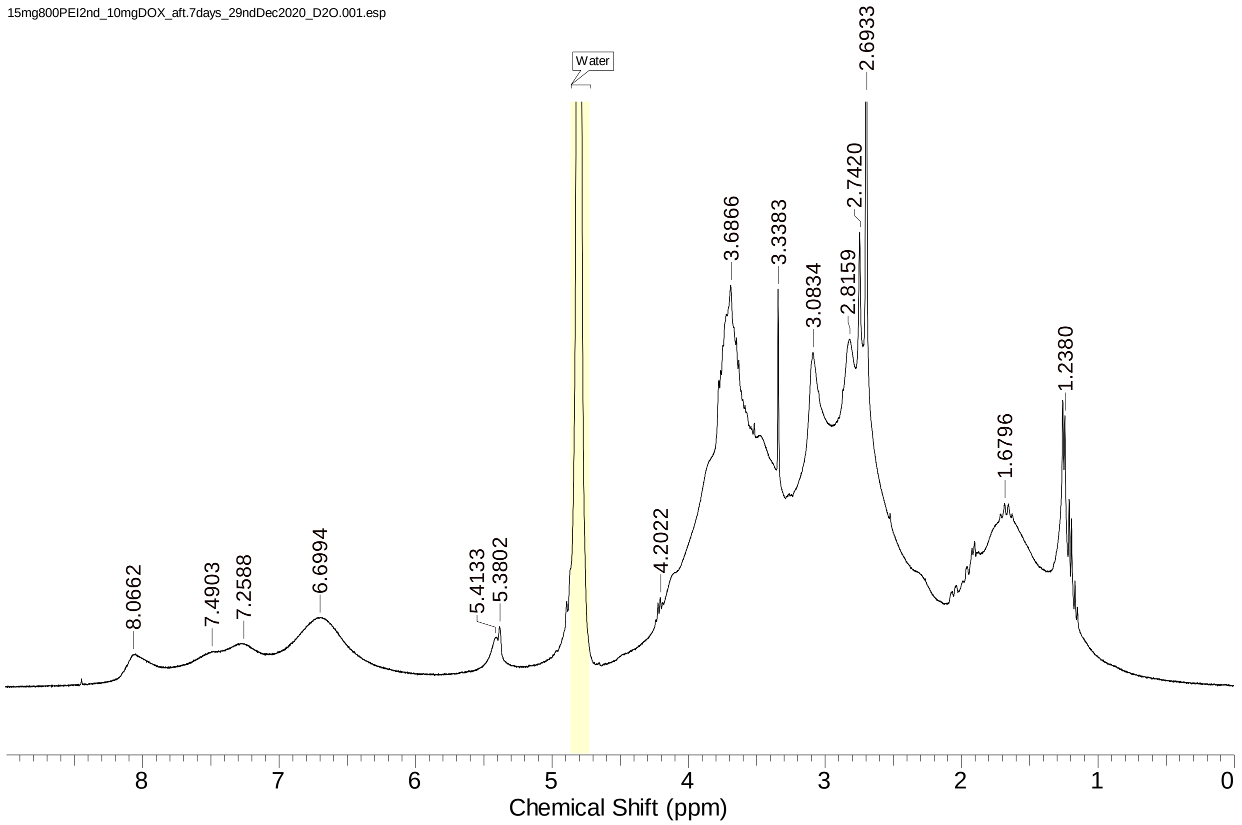

Figure S7. ^{1}H-NMR spectrum in D_2O, 0-9 ppm ranged**,** of **Cross-PEI-800** mixed with Doxorubicin after 1 week.

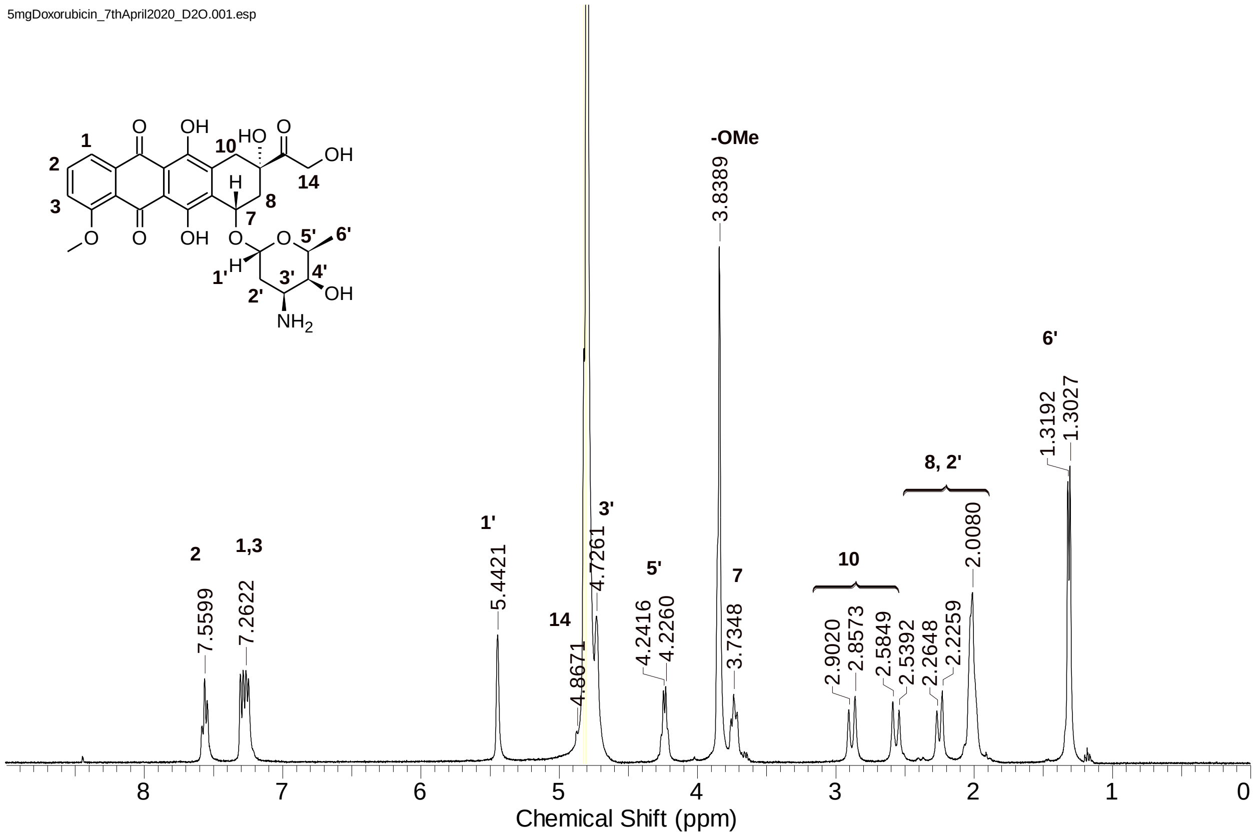

Figure S8. ^{1}H-NMR spectrum in D_2O, 0-9 ppm ranged, of Doxorubicin with peak assignament.

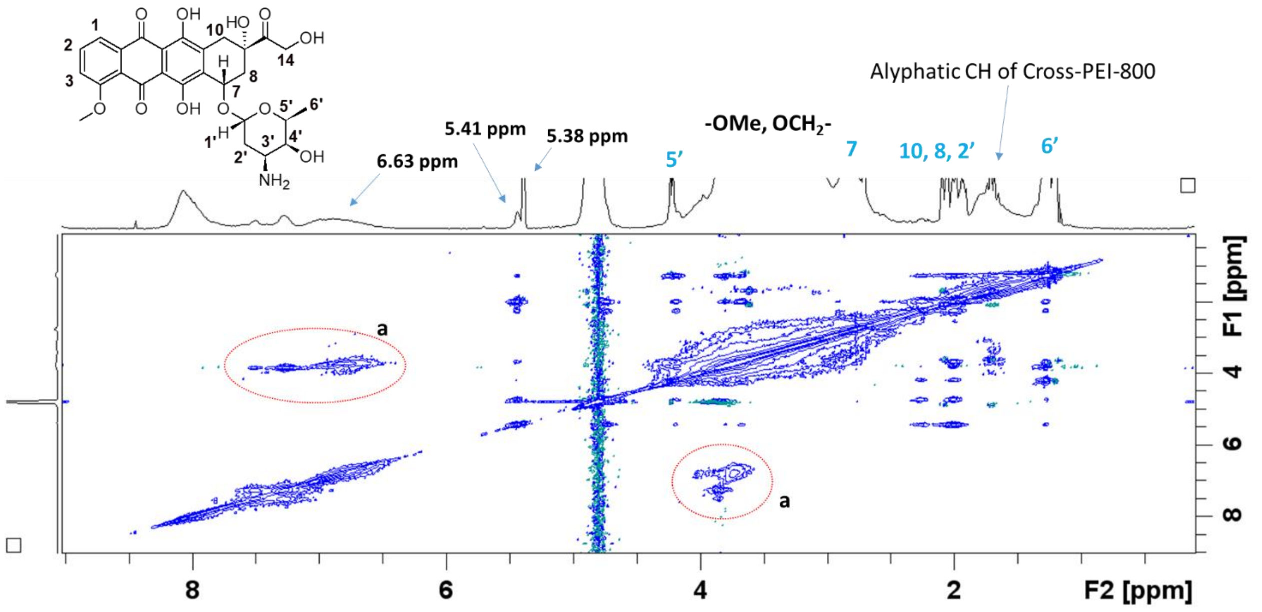

Figure S9. NOESY spectrum of doxorubicin mixed with **Cross-PEI-800**. The dash red line marked with a refer to the interactions of aromatic signals with –OMe, -OCH_2- signals.

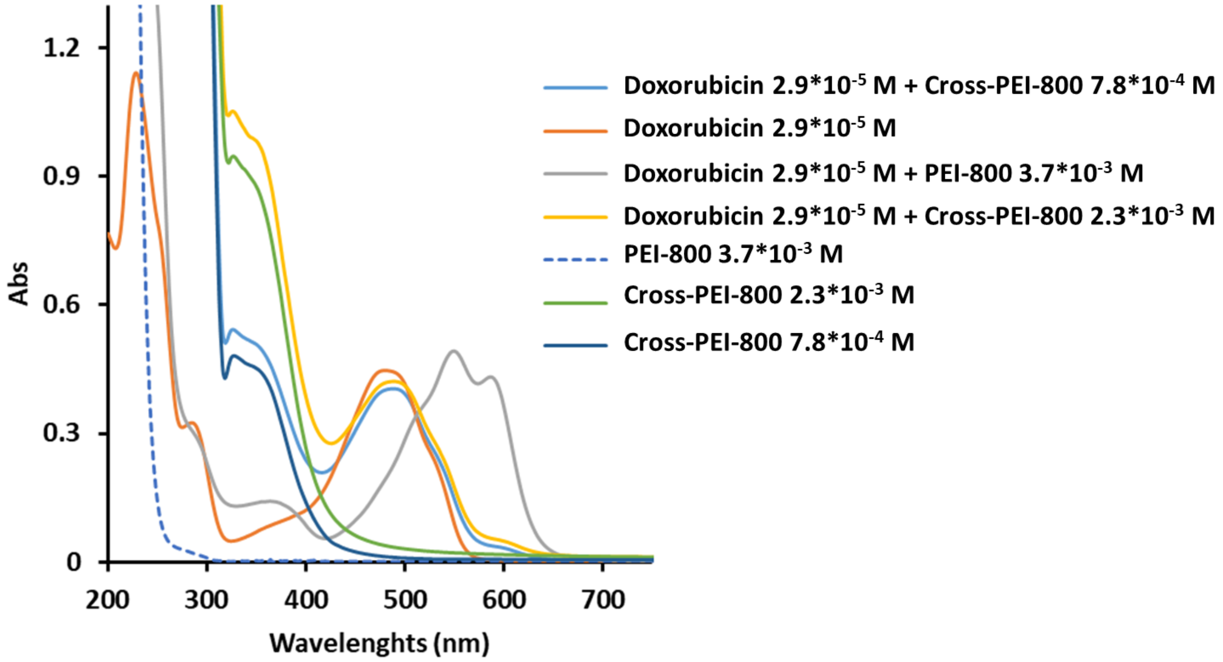

Figure S10. UV-Vis spectra of Doxorubicin (DOX), **PEI-800**, **Cross-PEI-800** in pure and mixed solutions. Character * is multiplication.

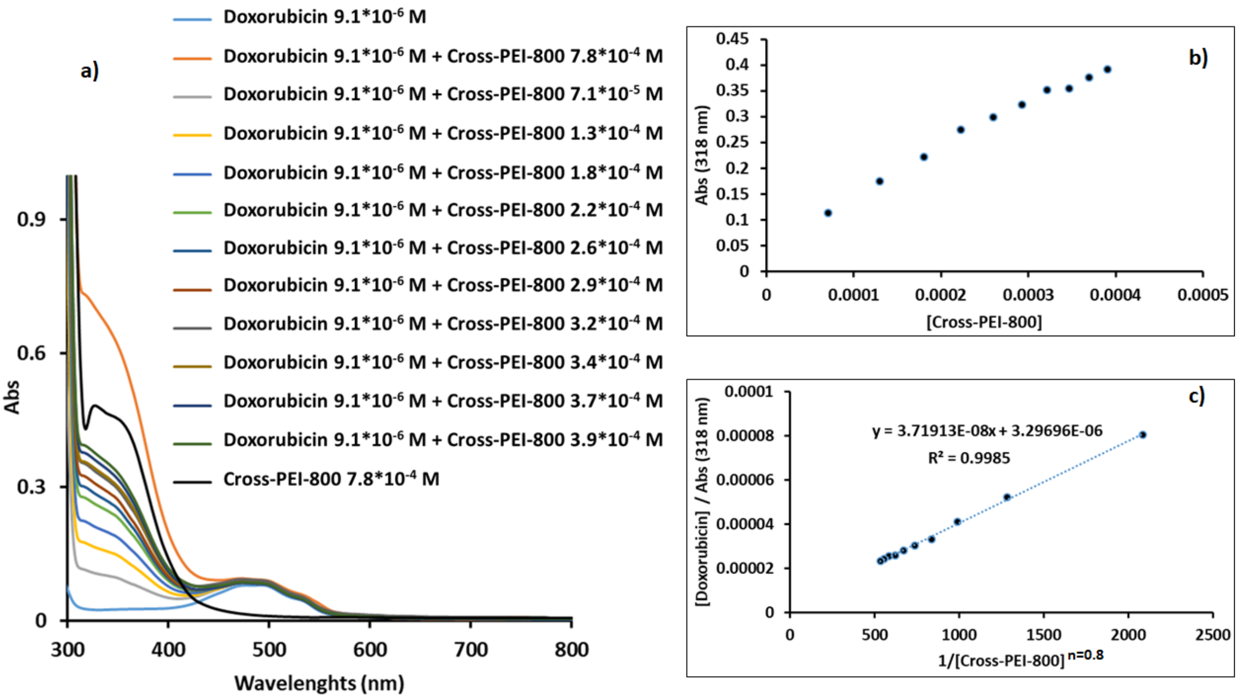

Figure S11. a) Titration of Doxorubicin with **Cross-PEI-800** (character * is multiplication). We elected drug and cross-linked polymer concentrations that during the titration maintain constant the Doxorubicin abs. Due to the cross-linked polymer and drug absorption overlap it is difficult to follow the diminution of the Doxorubicin abs by titration. The growing of abs absorption at 318 nm mainly depends on host guest interactions because the pure **Cross-PEI-800** absorption at this frequency displays a minimum and pure Doxorubicin does not absorbs. M if referred to CTV molar units for mL. We titrated the Doxorubicin 9.1x10-6 M solution with a solution of Doxorubicin 9.1x10^{-6} M + **Cross-PEI-800** 7.8x10^{-4} M (in this way we added the cross-linked polymer maintaining constant the Doxorubicin concentration). b) The variation of absorption as function of

the CTV concentration. c) If [Cross-PEI-800] and [Dox] are respectively the concentrations of CTV units and Doxorubicin, Dox interacts with n CTV units for obtaining the complex Dox(Cross-PEI-800)$_n$, with Keq as equilibrium constant. If Abs is the absorbance variation during the titrations, we can write the following relationships:

$$\mathrm{Keq} = \frac{[\mathrm{Dox(Cross{-}PEI{-}800)_n}]}{[\mathrm{Cross{-}PEI{-}800}]^n\,[\mathrm{Dox}]}$$

$$\mathrm{Keq} \propto \frac{\mathrm{Abs}}{[\mathrm{Cross{-}PEI{-}800}]^n[Dox]} \Rightarrow \frac{[\mathrm{Dox}]}{\mathrm{Abs}} \propto \frac{1}{\mathrm{K_{eq}}[\mathrm{Cross{-}PEI{-}800}]^n}$$

The function [Dox]/Abs vs 1/[Cross-PEI-800]^n is a straight line with the optimal n value. The optimal n represents the stoichiometry of the interaction between Doxorubicin and CTV. If we consider the Abs of the maximum at 318 nm, the best value of n is 0.8 and the constant is 2.7×10^7.

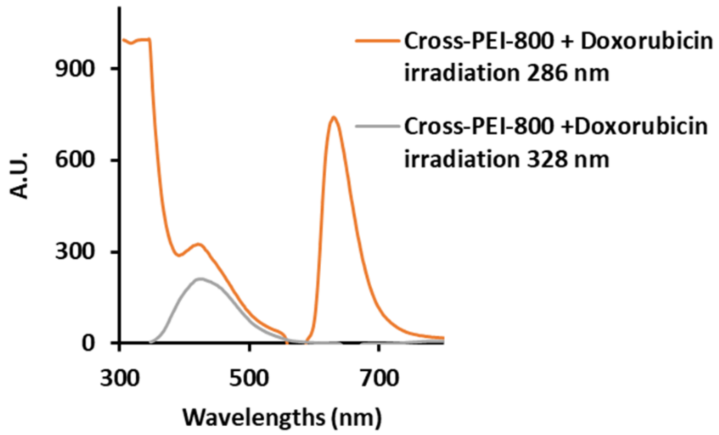

Figure S12. Fluorescence spectra of the Doxorubicin (5.7×10^{-6} M) mixed with **Cross-PEI 800** (1.5×10^{-4} M) irradiated at the frequencies of absorption maxima of the cross-linked polymer.

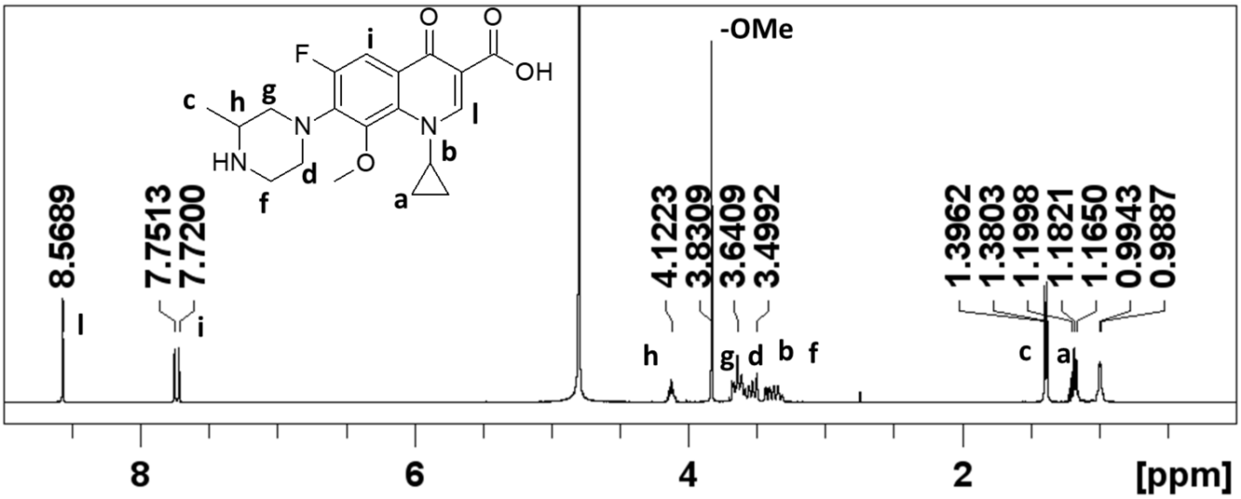

Figure S13. ^{1}H-NMR spectrum in D_2O of Gatifloxacin**,** the signals assigned as reported in literature (Ebtehal S. Al-Abdullah, in 'Profiles of Drug Substances, Excipients and Related Methodology', 2012).

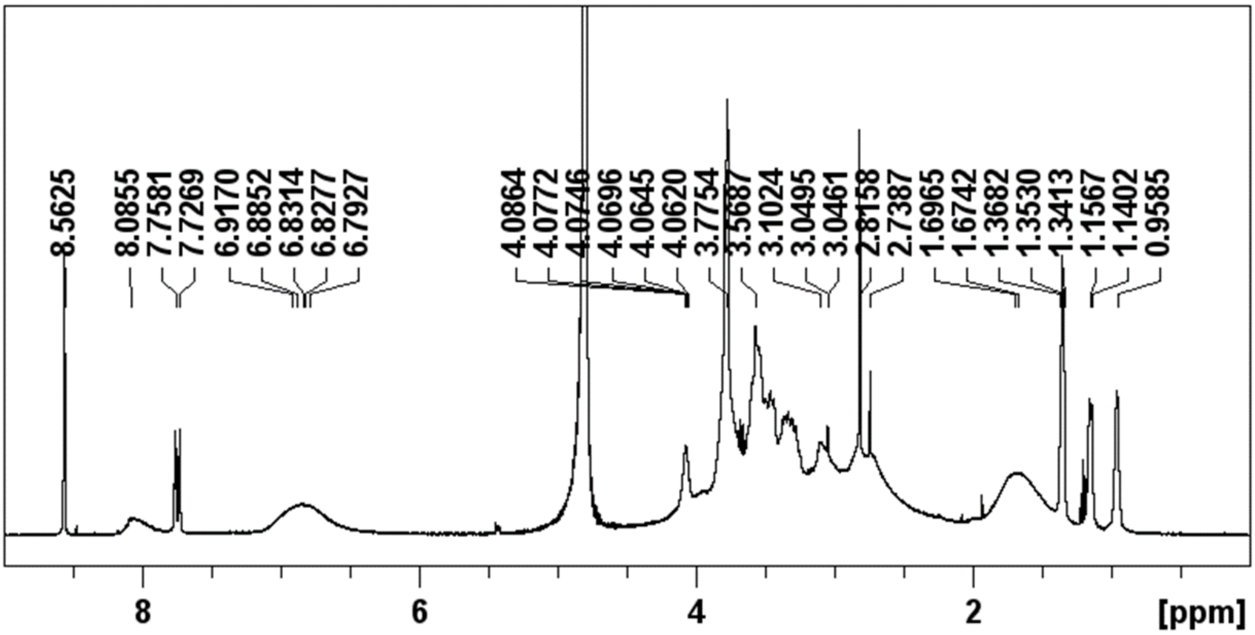

Figure S14. ^{1}H-NMR spectrum of **Cross-PEI-800** mixed with Gatifloxacin (5 mg + 5 mg).

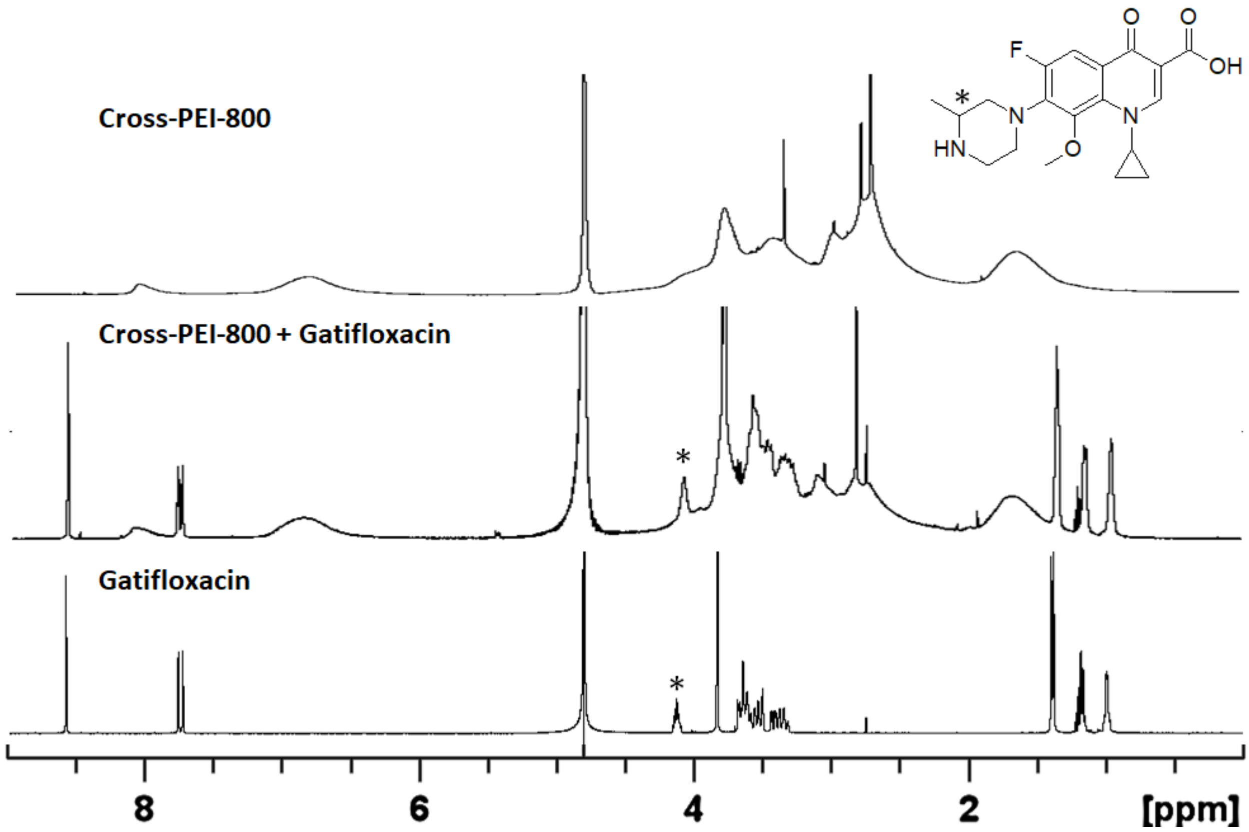

Figure S15. Comparation between ^{1}H-NMR spectra of Gatifloxacine, **Cross-PEI-800,** Gatifloxacine + **Cross-PEI-800.** The signal marked with * is the signal indicated in the structure how reported in literature (Ebtehal S. Al-Abdullah, in 'Profiles of Drug Substances, Excipients and Related Methodology', 2012).

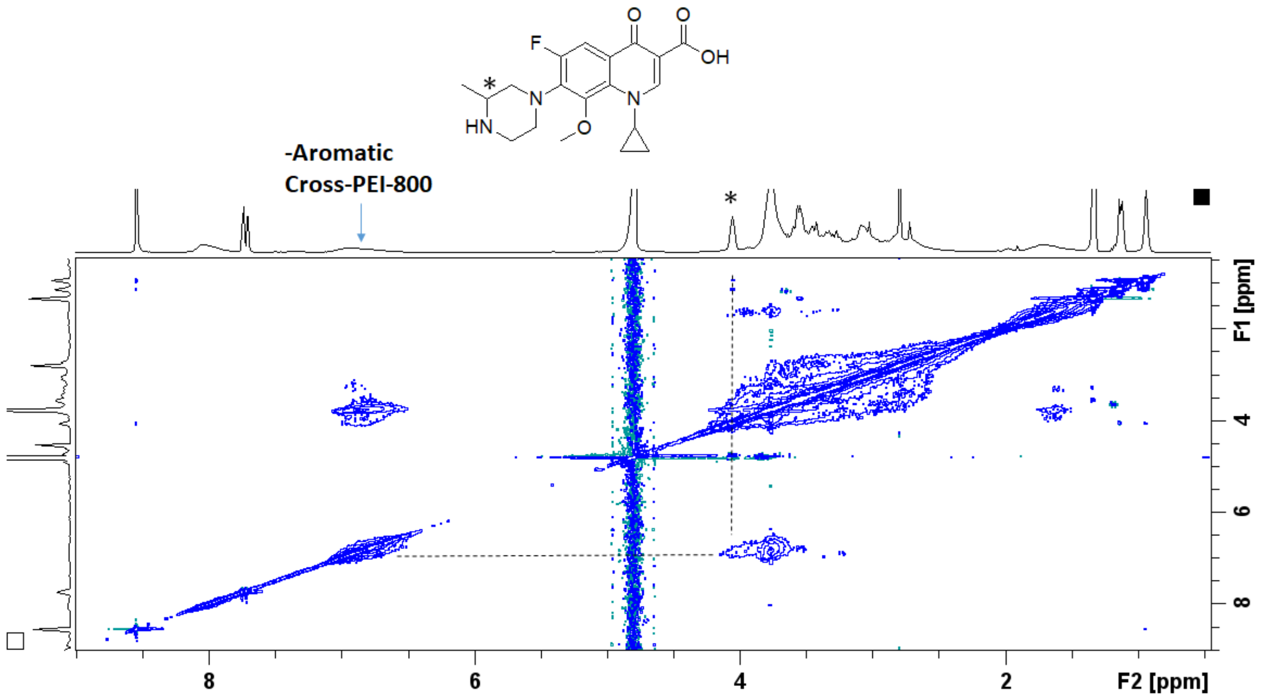

Figure S16. NOESY spectrum of Gatifloxacin mixed with **Cross-PEI-800**.

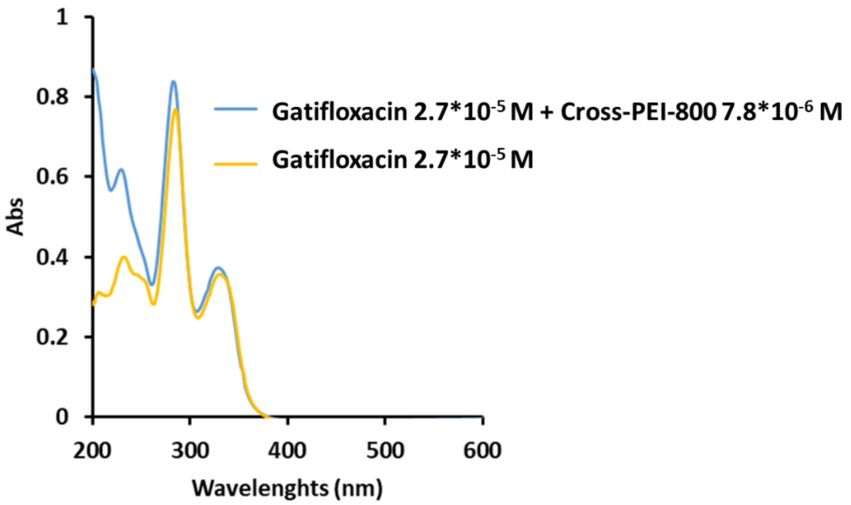

Figure S17. UV-Vis spectra of Gatifloxacin with and without crosslinked polymer.

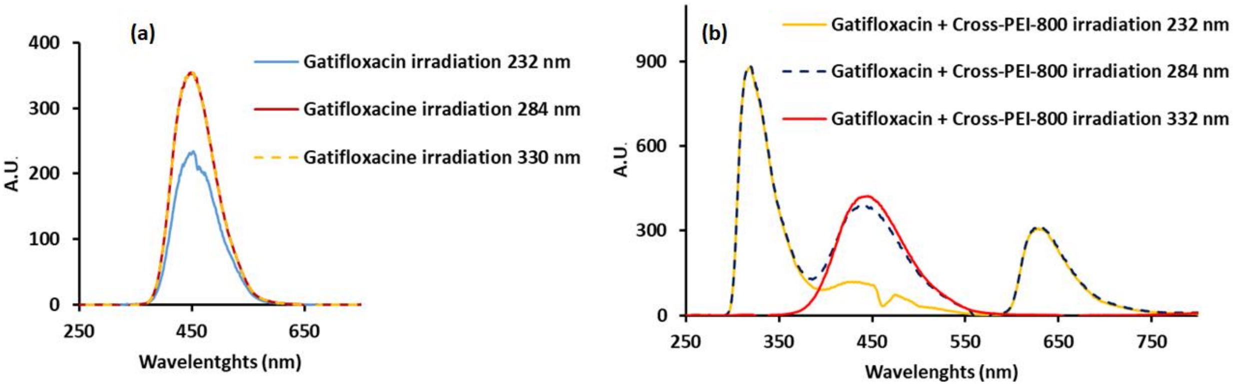

Figure S18. Fluorescence spectra of a) Gatifloxacine $5.3x10^{-6}$ M, b) Gatifloxacine $5.3x10^{-6}$ M mixed with **Cross-PEI-800** $1.6x10^{-4}$ M.

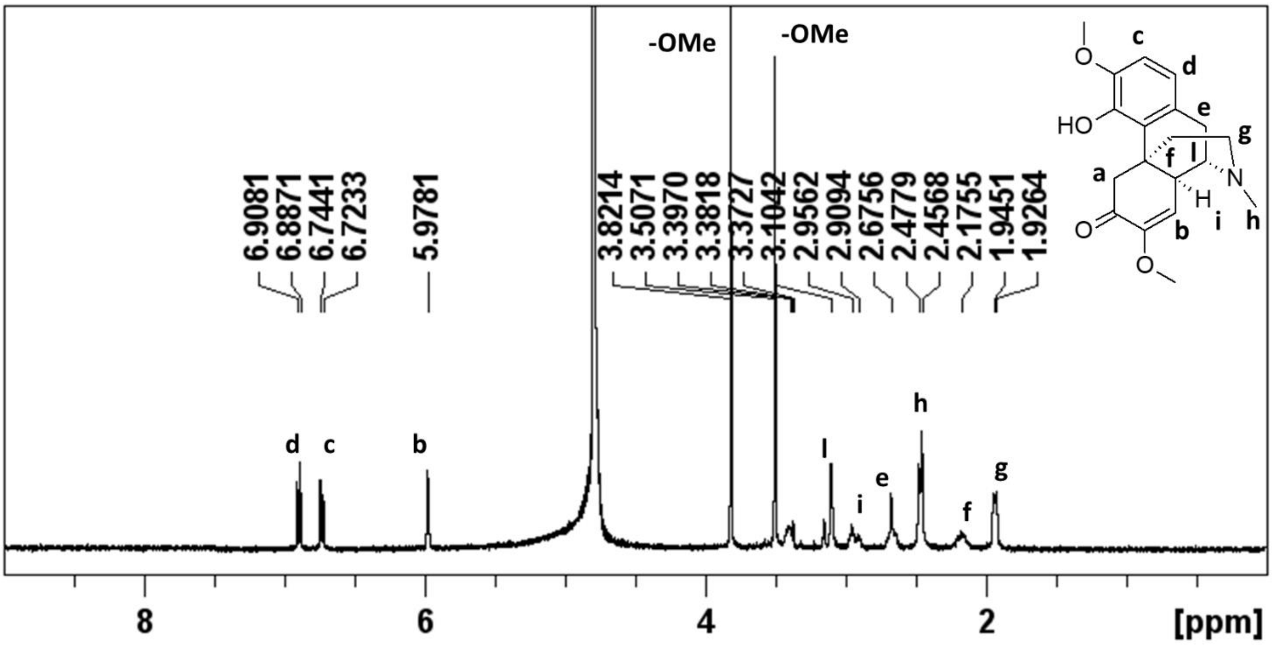

Figure S19. ^{1}H-NMR spectrum in D_2O of Sinomenine. We assigned the 1HNMR signals basing on the reported literature data about the NMR spectra in DMSO-d_6.

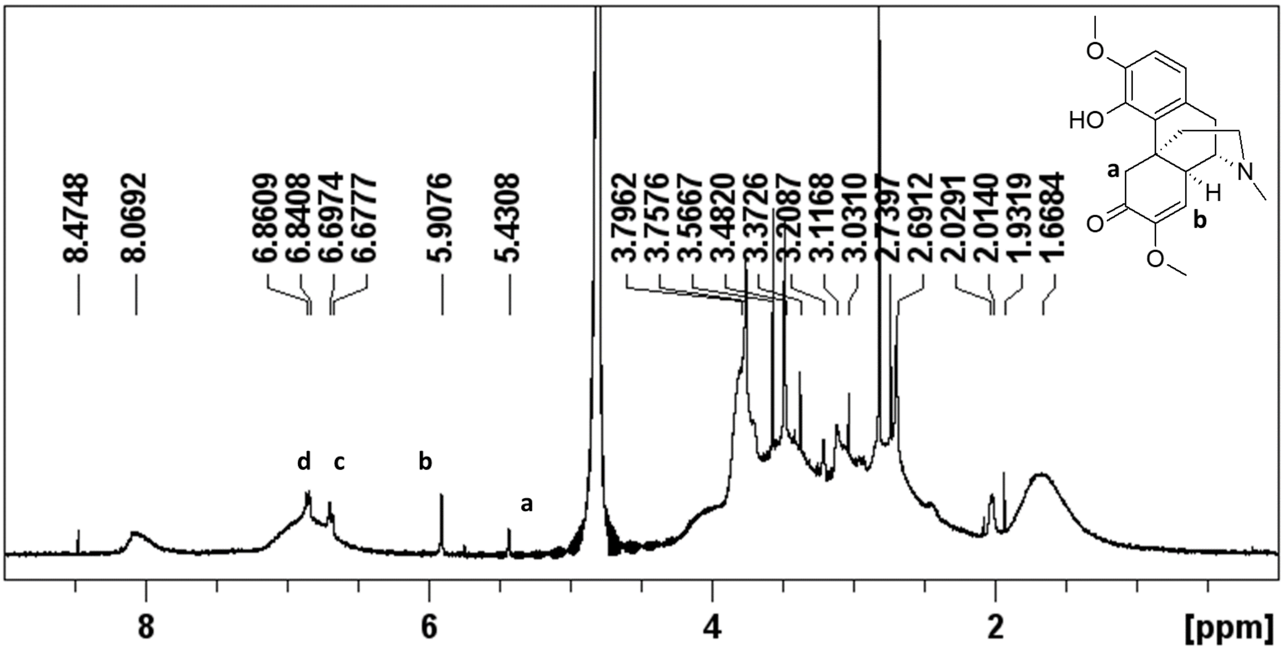

Figure S20. ^{1}H-NMR spectrum of Sinomenine in the presence of **Cross-PEI-800** (5mg + 5mg).

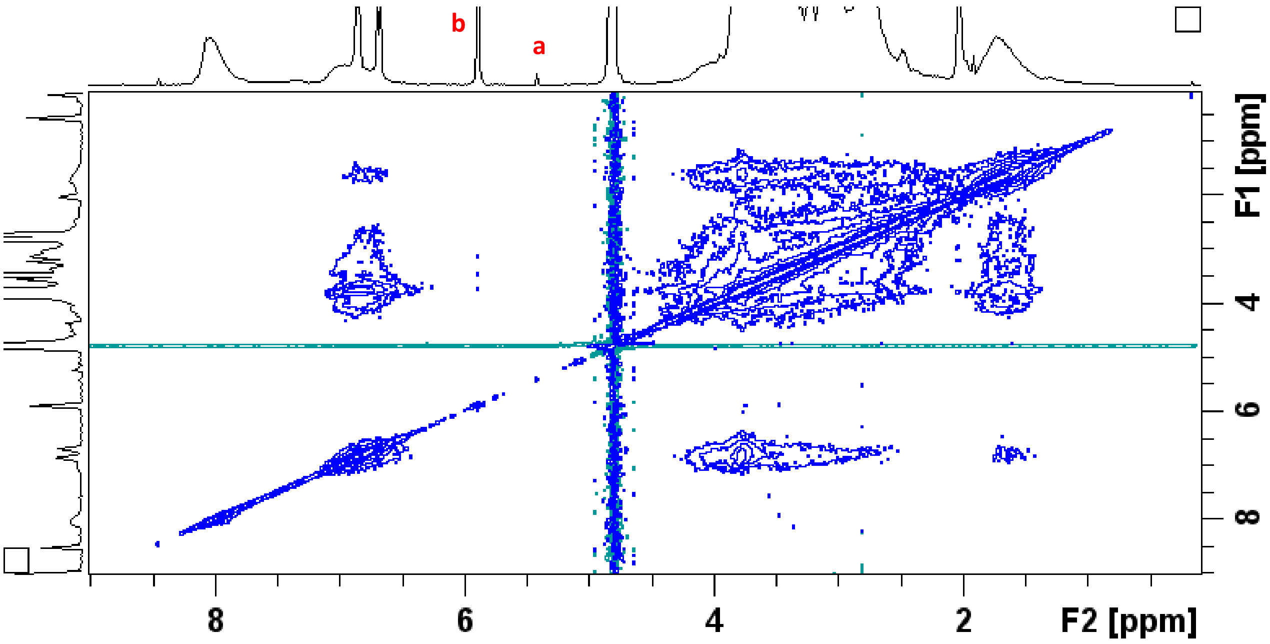

Figure S21. NOESY spectrum of Sinomenine in the presence of Cross-PEI-800 (5mg + 5mg). It is difficult to detect the interactions between the cross-linked polymer and Sinomenine because the aromatic signals of the two molecules lay in the same ppm range. The b signal exhibits some interaction with the –OMe signals and it is lowered shifted, it can prove some hydrophobic interactions with the armed CTV.

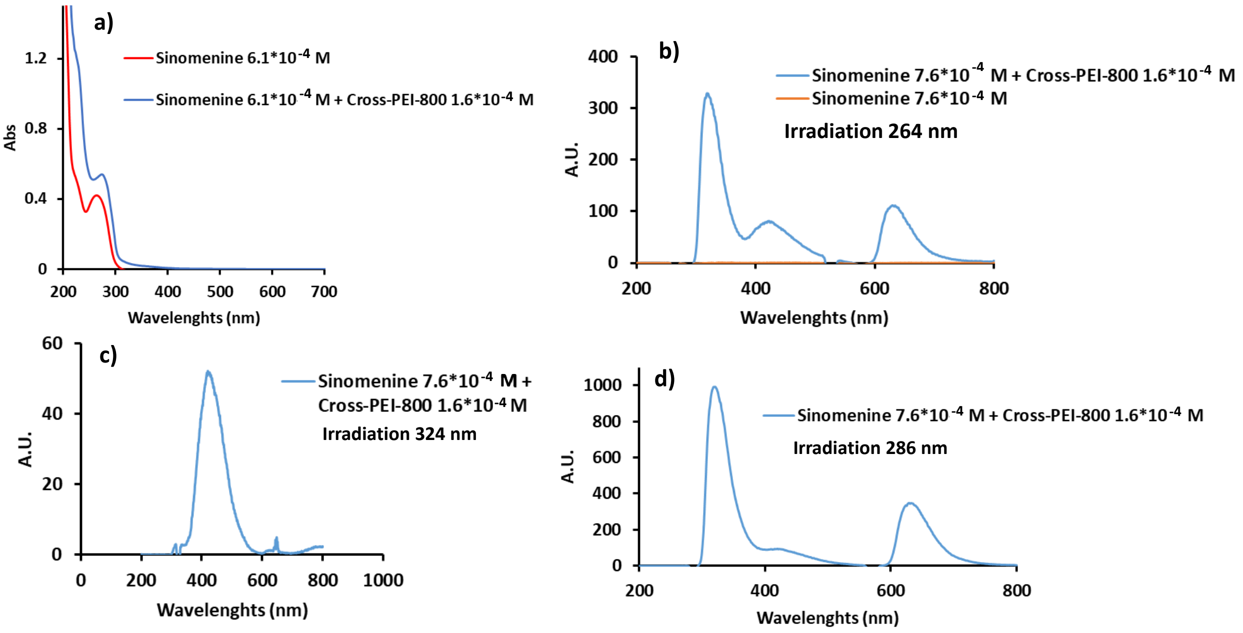

Figure S22. a) UV-vis spectra of SIN pure and in the presence of **Cross-PEI-800**. b) Emission spectrum of SIN pure and in the presence of **Cross-PEI-800**, irradiation at 264 nm. c) Emission spectrum of SIN in the presence of **Cross-PEI-800**, irradiation at 324 nm. d) Emission spectrum of SIN in the presence of **Cross-PEI-800**, irradiation at 286 nm.

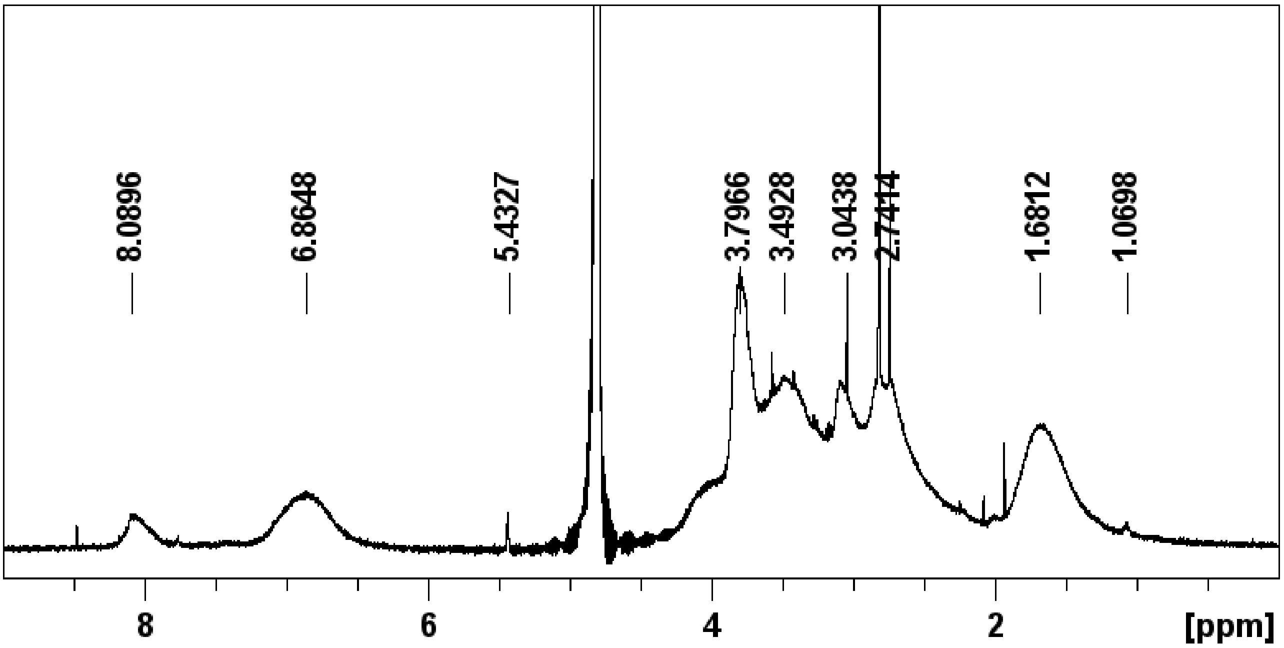

Figure S23. ^{1}H-NMR spectrum of Camptothecine in the presence of **Cross-PEI-800** (5mg + 5mg), range 0-9 ppm.

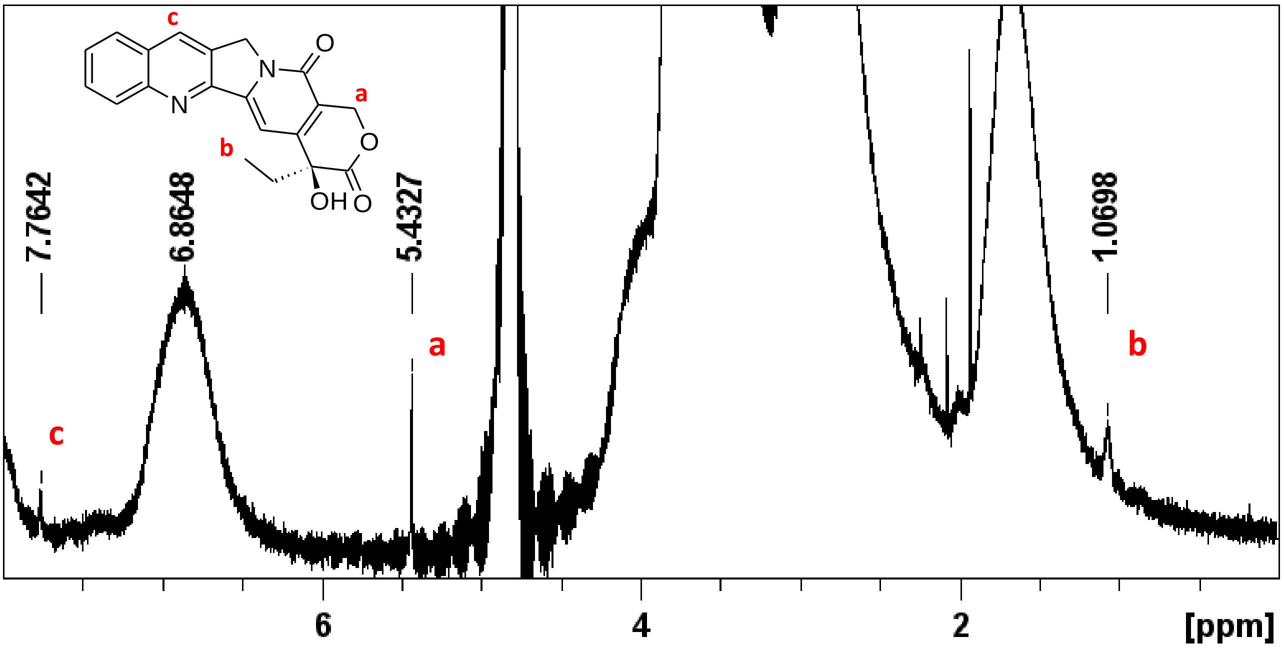

Figure S24. ^{1}H-NMR spectrum of Camptothecine in the presence of **Cross-PEI-800** (5mg + 5mg), range 0-8 ppm with enlarged signals and assignations.

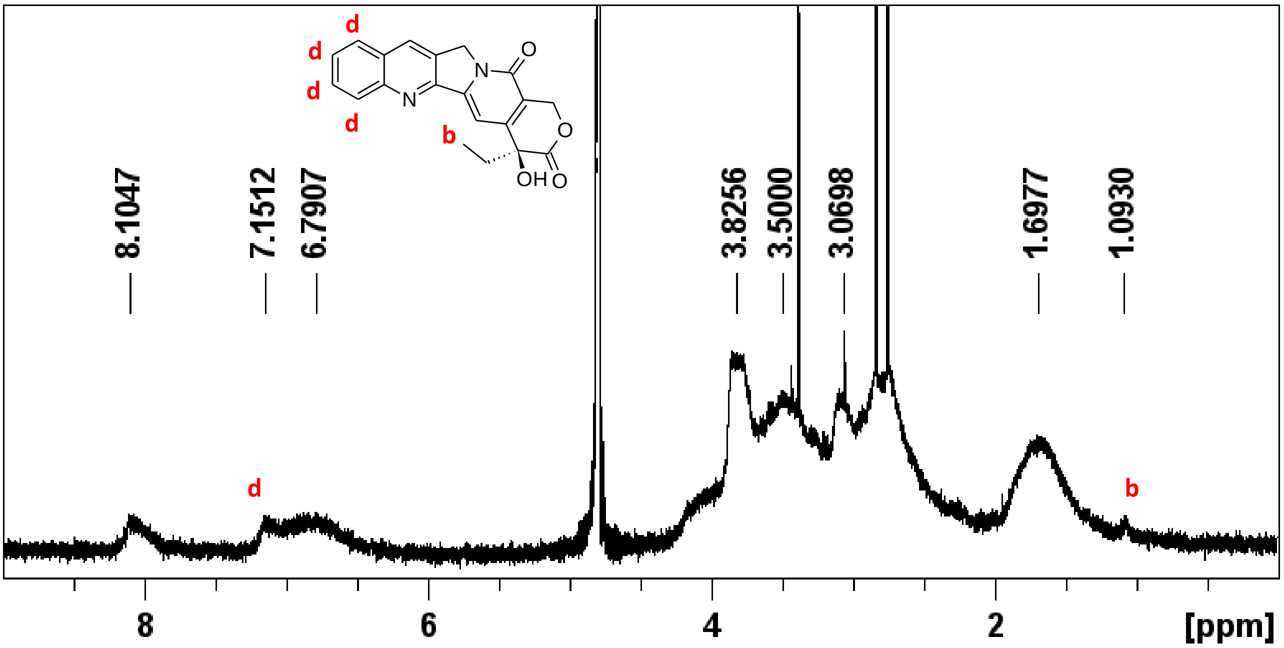

Figure S25. ^{1}H-NMR spectrum of Camptothecine in the presence of **Cross-PEI-800** (2 mg crosslinked polymer + 7 mg of drug), range 0-9 ppm with assignation of the aromatic and aliphatic signals.

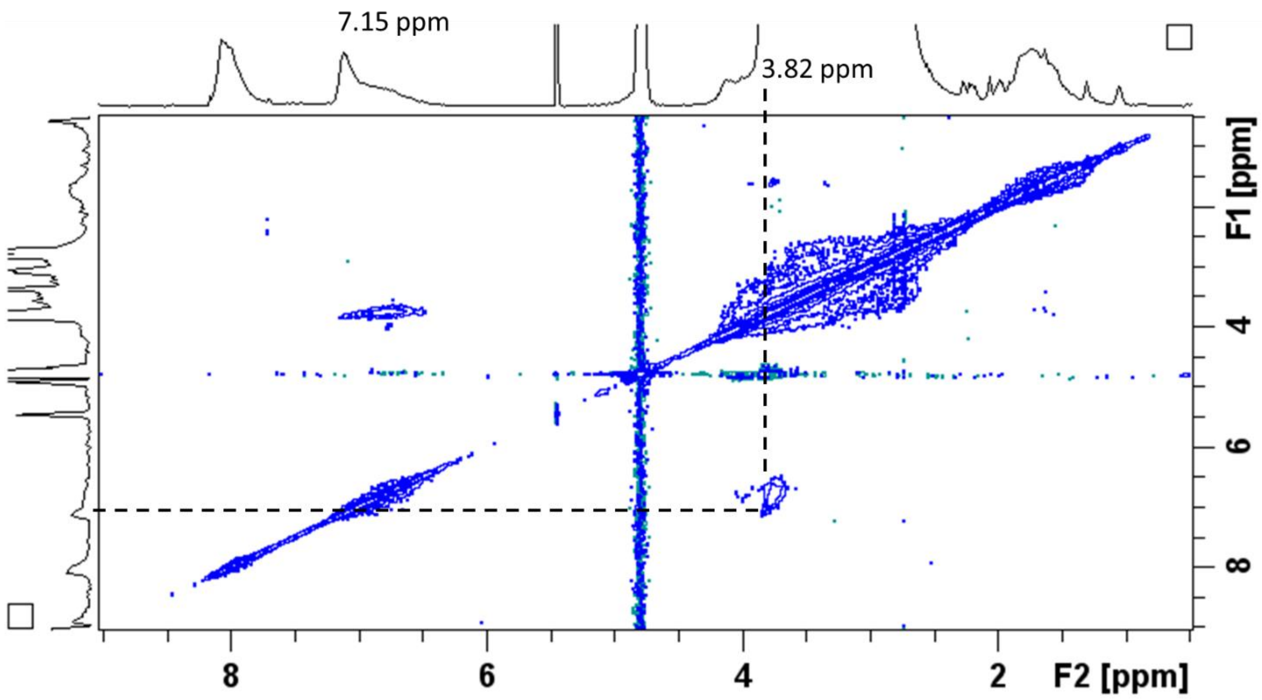

Figure S26. NOESY spectrum of Camptothecine in the presence of **Cross-PEI-800** (2 mg crosslinked polymer + 7 mg of drug).

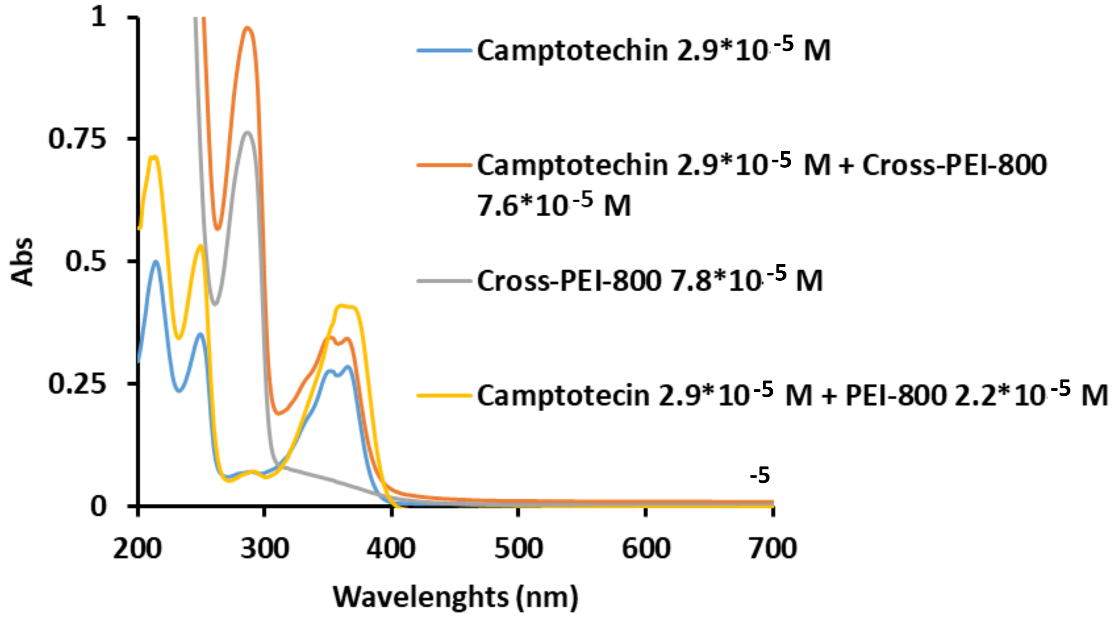

Figure S27. UV-Vis spectra of Camptothecin pure and in the presence of **Cross-PEI-800, PEI-800**.

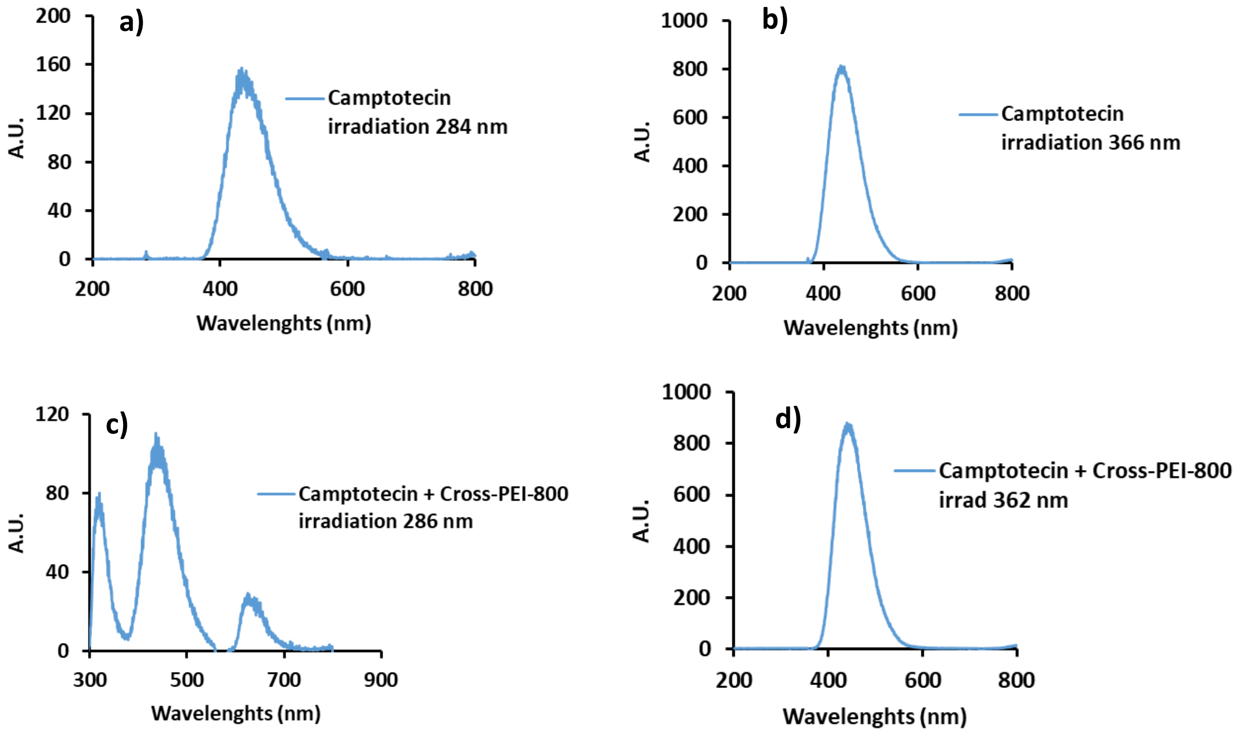

Figure S28. Emission spectra of pure Camptotechin 1.4x10^{-3} M irradiated at a) 284 nm, b) at 366 nm and in the presence of **Cross-PEI-800** 1.6x10^{-4} M irradiated at c) 286 nm, d) 362 nm.

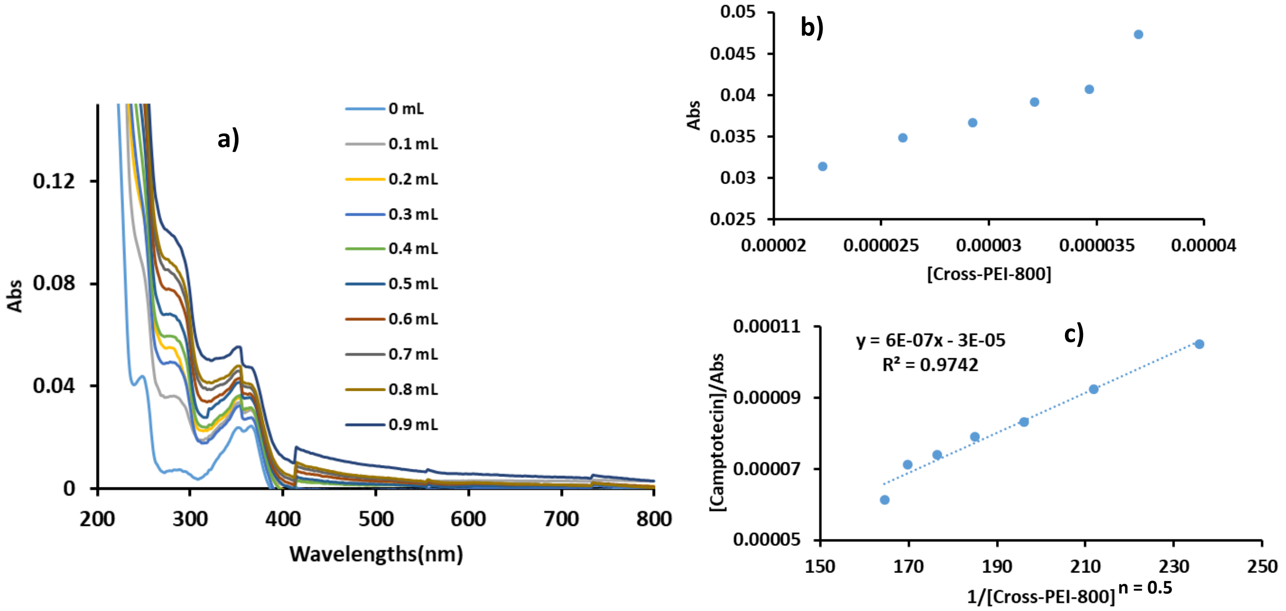

Figure S29. Titration of Camptotechin solution 2.9x10^{-5} M with a solution of **Cross-PEI-800** 7.8x10^{-5} M (character * is multiplication) followed by UV-vis absorption spectroscopy. a) UV-vis spectra during the titration, b) Absorption maximum of Camptotecin at 366 nm at different **Cross-PEI-800** concentrations, c) The function [Camptothecin]/Abs vs 1/[Cross-PEI-800]^n is a straight line where the optimal n value represents the stoichiometry of the interaction between Camptothecin and CTV. The optimal value of n is 0.5 and the constant is 2x10^6.

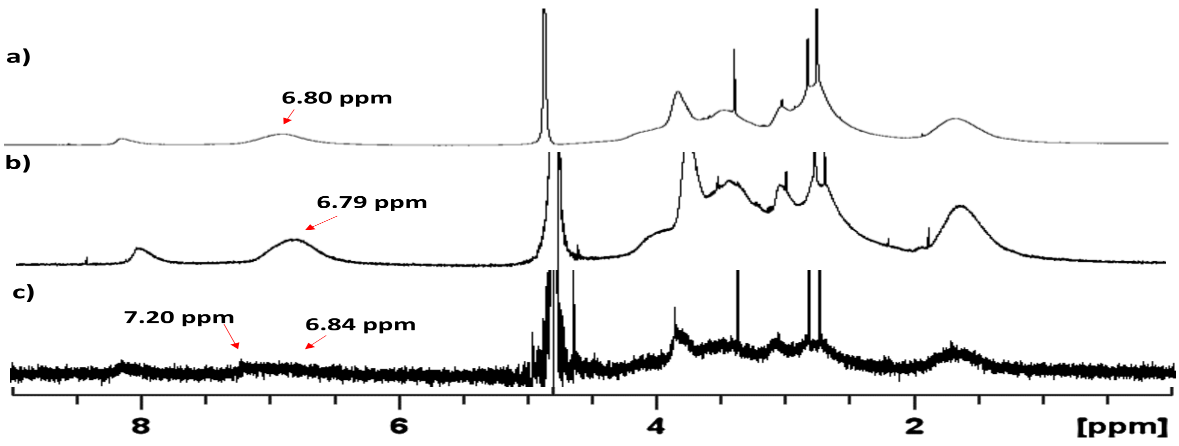

Figure S30. ^{1}H-NMR spectra of a) **Cross-PEI-800,** Celastrol (5mg) in the presence of **Cross-PEI-800** (5mg) and, c) Celastrol (7 mg) in the presence of **Cross-PEI-800** (2 mg).

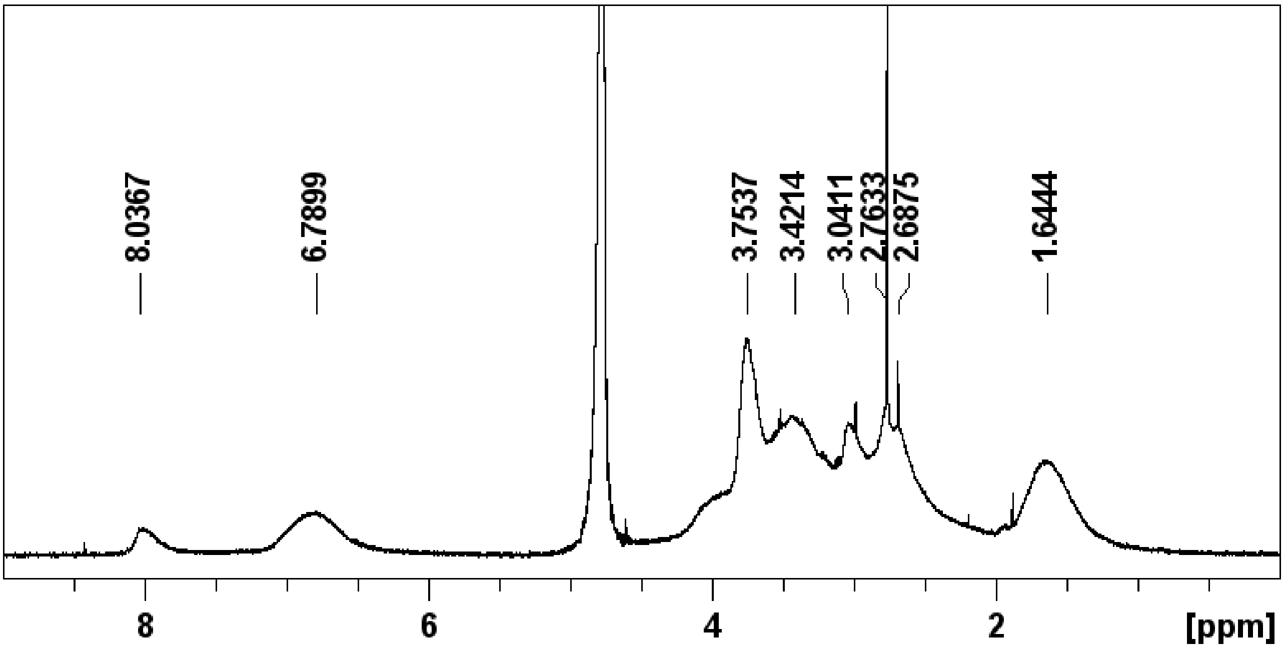

Figure S31. ^{1}H-NMR spectrum of Celastrol (5mg) in the presence of **Cross-PEI-800** (5mg).

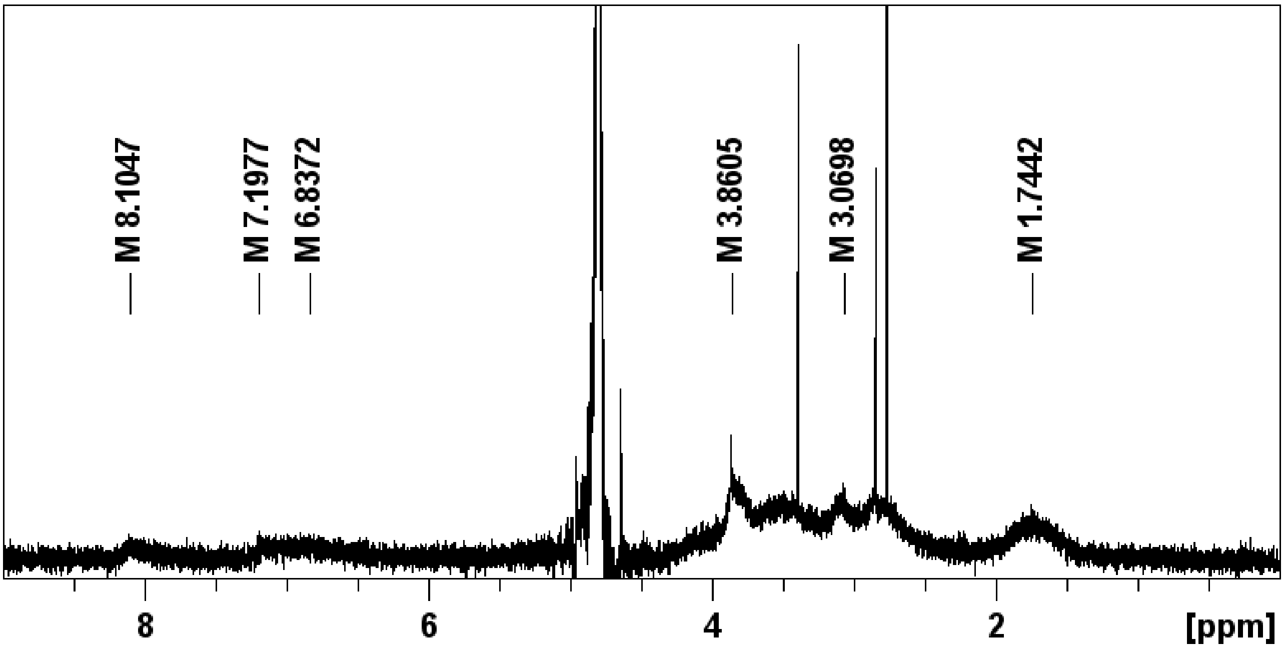

Figure S32. ^{1}**H-NMR** of Celastrol (7 mg) in the presence of **Cross-PEI-800** (2 mg).

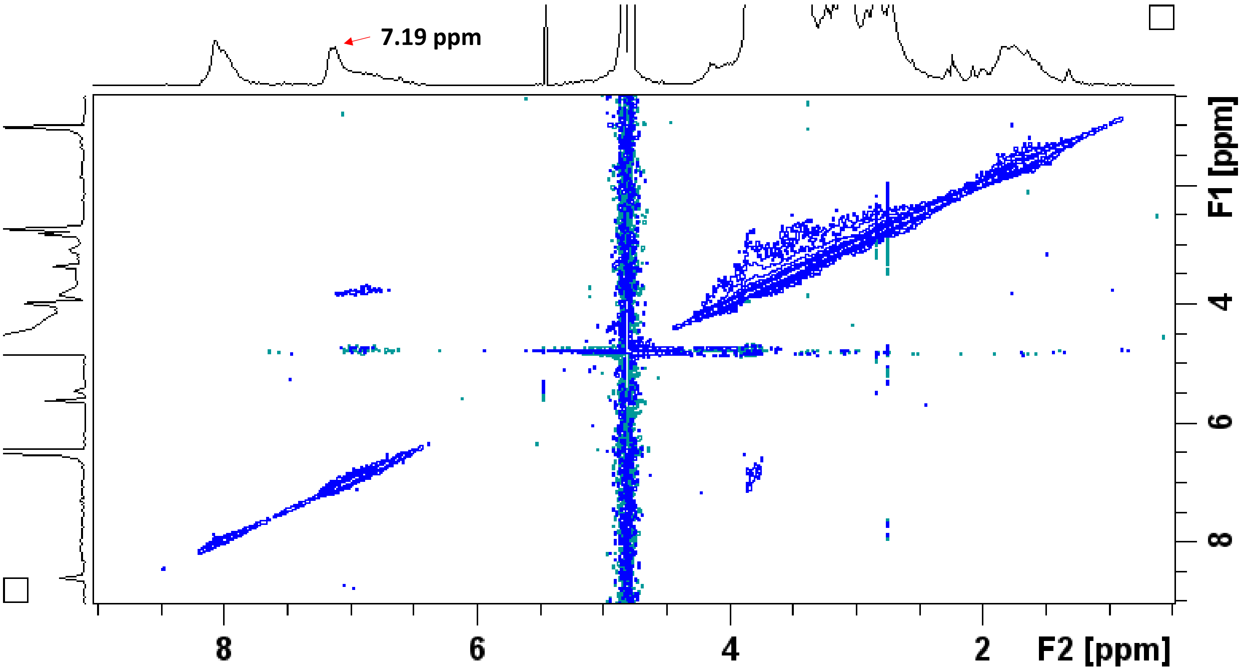

Figure S33. NOESY spectrum of Celastrol (7 mg) in the presence of **Cross-PEI-800** (2 mg).

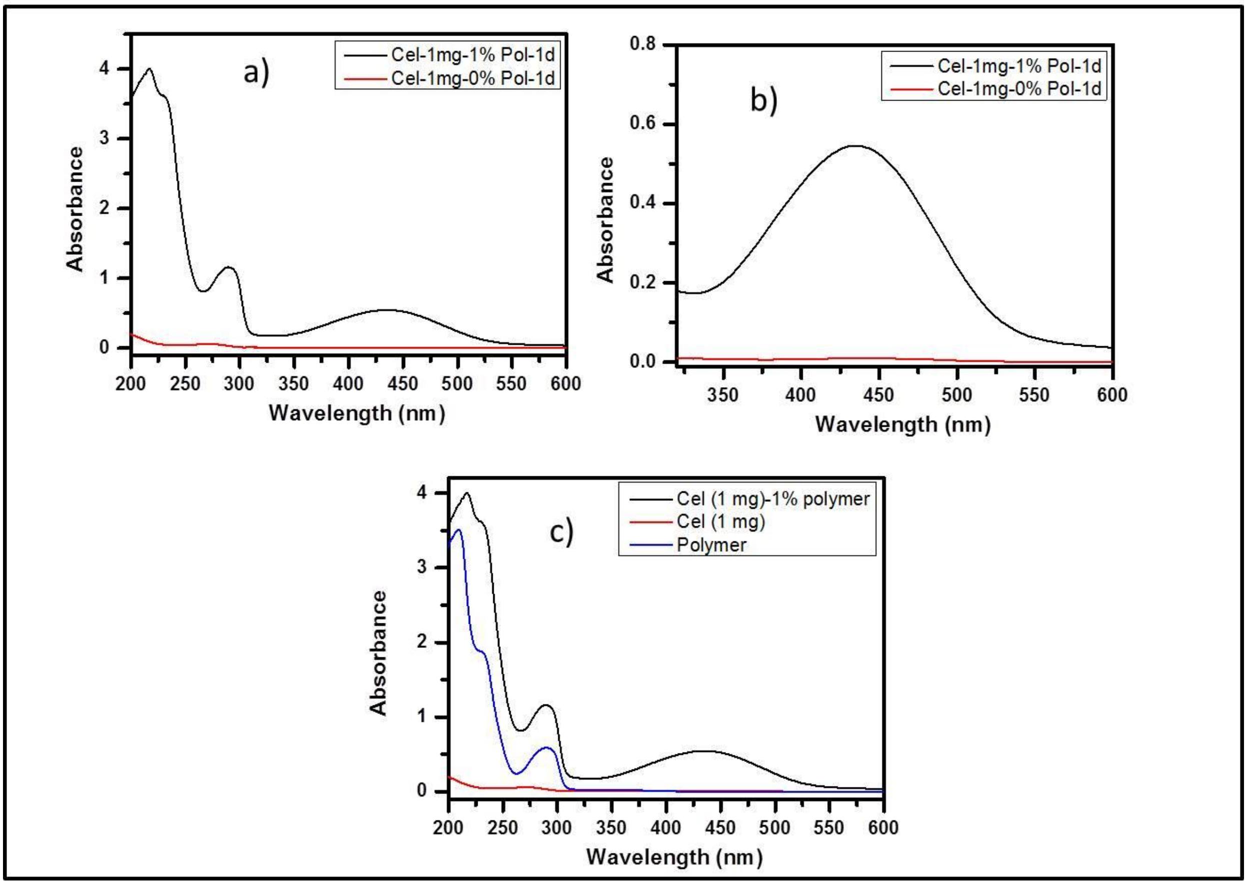

Figure S34. a) UV-Vis spectrum of solution of Celastrol 2.2x10^{-3} M pure (red line) and in the presence of 1% of polymer **Cross-PEI-800** (black line), b) Magnification spectra among 300-600nm, c) Comparison spectra polymer pure (blue line), solution of Celastrol (red line) and Celastrol-polymer1%.

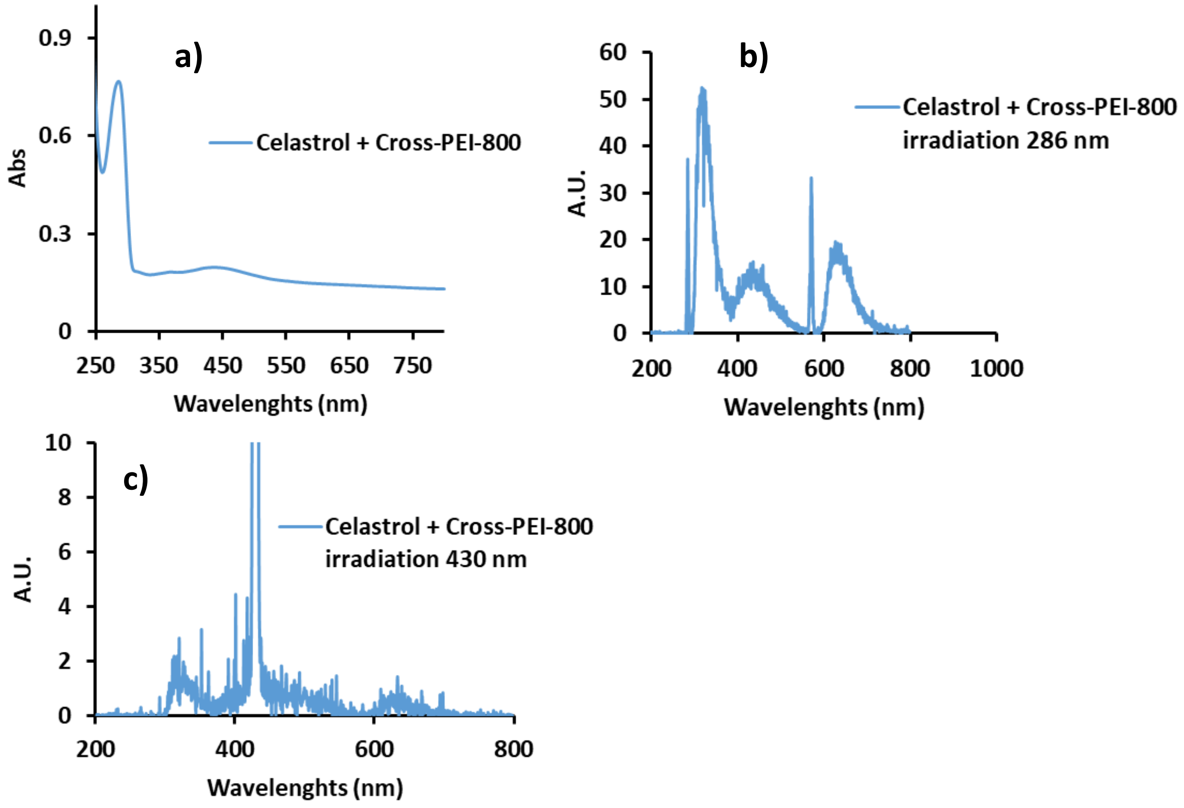

Figure S35. Solution of Celastrol 1.1x10^{-3} M in the presence of **Cross-PEI-800** 7.8x10^{-5} M, a) UV-absorption spectrum, b) emission spectrum upon irradiation at 286 nm, c) emission spectrum upon irradiation at 430 nm.

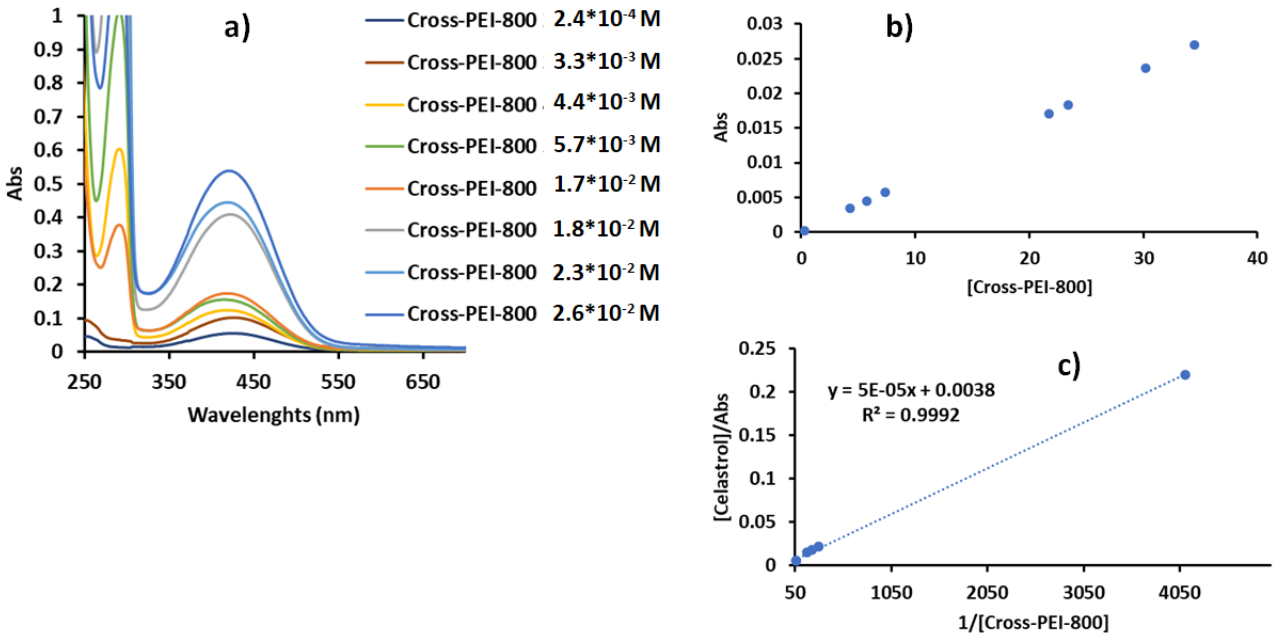

Figure S36. Titration of Celastrol solution 2.2x10^{-3} M with a solution of **Cross-PEI-800** 7.8x10^{-5} M followed by UV-vis absorption spectroscopy. a) UV-vis spectra during the titration, b) Absorption maximum of Celastrol at 435 nm at different **Cross-PEI-800** concentrations, c) The function

[Celastrol]/Abs vs 1/[Cross-PEI-800]^n is a straight line where the optimal n value represents the stoichiometry of the interaction between Celastrol and CTV. The optimal value of n is 1 and the constant is $2x10^5$.

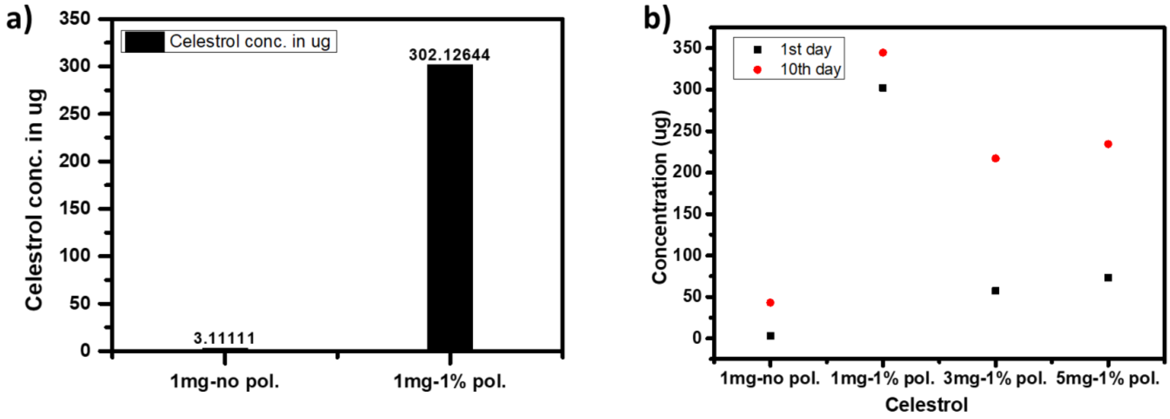

Figure S37. a) Water solubility of Celastrol with and without addition of Cross-PEI-800. b) Solubility variation after 10 days.

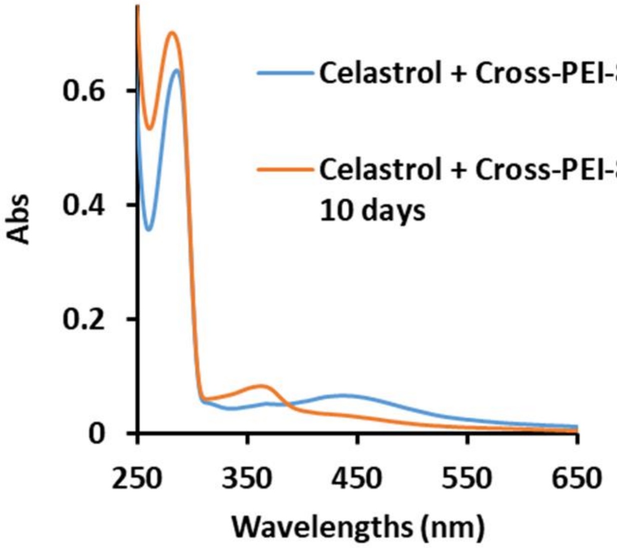

Figure S38. UV-vis spectra of Celastrol $1.1x10^{-3}$ M pure and mixed with **Cross-PEI-800** $7.8x10^{-5}$ M, soon after mixing and after 10 days.

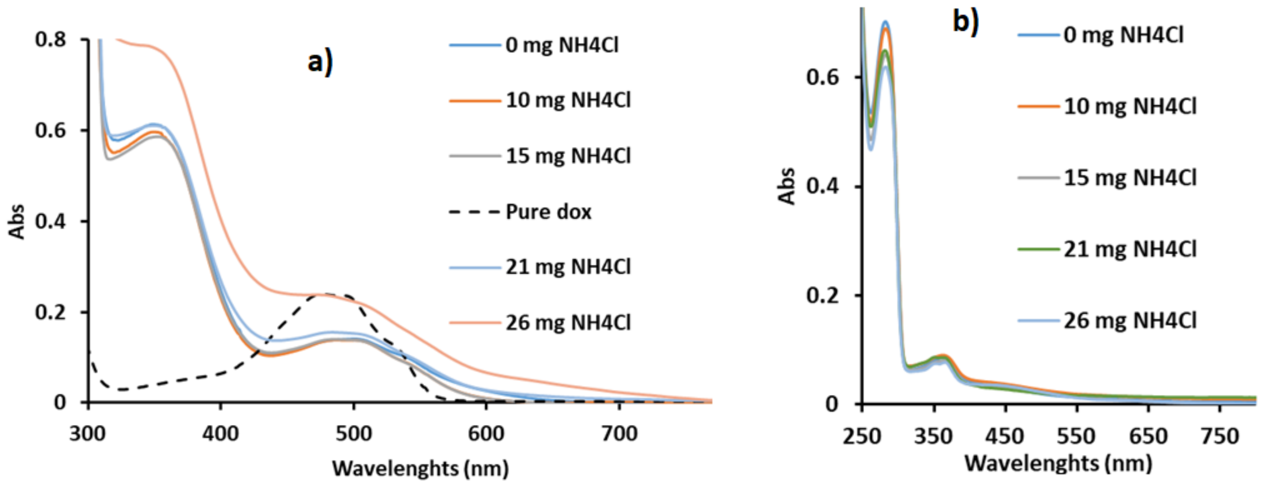

Figure S39. a) UV-vis spectra of Doxorubicin $2.9x10^{-5}$ M mixed with **Cross-PEI-800** $7.8x10^{-4}$ M and with NH_4Cl addition. b) UV-vis spectra of Celastrol $1.1x10^{-3}$ M mixed with **Cross-PEI-800** $7.8x10^{-5}$ M and with NH_4Cl addition. During the NH_4Cl addition, the solution pH remained 7.

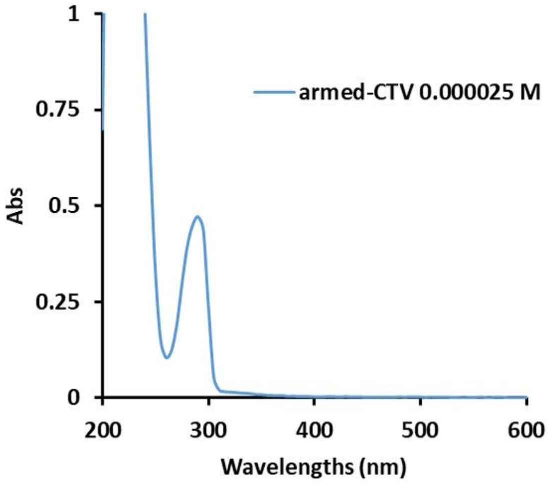

Figure S40. UV-vis spectrum of **Armed-CTV** $2.5x10^{-5}$ M in Methanol. This compound, synthetic precursor of Cross-PEI-800 is not soluble in water.

Table S1. Cytotoxicity of polymer Cross-PEI-800 against normal and cancer cell lines

**Compound**	A549 cells IC_{50}[µg/mL]	LO2 cells IC_{50}[µg/mL]	Hek293 cells IC_{50}[µg/mL]
Cross-PEI-800	**>500**	**59.314**	**321.59**

Abbreviations

DOX doxorubicin

SIN sinomenine

CEL celastrol
